# Anti-hyperglycemic activity of myricetin, through inhibition of DPP-4 and enhanced GLP-1 levels, is attenuated by co-ingestion with lectin-rich protein

**DOI:** 10.1371/journal.pone.0231543

**Published:** 2020-04-13

**Authors:** Nanjaiah Lalitha, Bettadahalli Sadashivaiah, Talahalli Ravichandra Ramaprasad, Sridevi Annapurna Singh

**Affiliations:** 1 Department of Protein Chemistry and Technology, CSIR- Central Food Technological Research Institute, Mysuru-, Karnataka, India; 2 Department of Biochemistry, CSIR- Central Food Technological Research Institute, Mysuru, Karnataka, India; Stellenbosch University, SOUTH AFRICA

## Abstract

Dipeptidyl peptidase-4 (DPP-4) is a proteolytic enzyme responsible for the rapid degradation of Glucagon-like peptide 1 (GLP-1) that is required for the secretion of insulin. DPP-4 also influences activation of node like receptor family, pyrin domain containing 3 (NLRP3) inflammasome under diabetic conditions. Although several polyphenols are reported for various bioactivities, they are consumed as part of the food matrix and not in isolation. Horsegram (*Macrotyloma uniflorum*) is a rich source of myricetin (Myr) (35 μg/g flour), reported for its anti-hyperglycemic effect. In this investigation, we aimed to study the effect of Myr, singly, and in the presence of co-nutrient horsegram protein (HP) on DPP-4 activity and its consequential impact on GLP-1, insulin, and NLRP3 inflammasome in high-fat diet and single low dose streptozotocin (STZ)-induced diabetic male Wistar rats. In diabetic control (DC), the activity of DPP-4 and its expression were higher compared to treated groups. The consequential decrease in the circulating GLP-1 levels in the DC group, but not treated groups, further indicated the effectiveness of our test molecules under diabetic conditions. Specifically, Myr decreased DPP-4 activity and its expression levels with enhanced circulating GLP-1 and insulin levels. Myr administration also resulted in a lessening of diabetes-induced NLRP3 inflammasome activation and enhanced antioxidant enzyme activities. HP also proved to be efficient in reducing elevated blood glucose levels and enhancing antioxidant enzyme activities. However, Myr, in the presence of HP as a co-nutrient, had diminished capacity to inhibit DPP-4 and, consequently, reduced potential in ameliorating diabetic conditions. Myr proved to be a potent inhibitor of DPP-4 *in vitro* and *in vivo*, resulting in enhanced circulating GLP-1 and insulin levels, thereby improving diabetic conditions. Though Myr and HP, individually ameliorate diabetic conditions, their dietary combination had reduced efficiency.

## Introduction

The proteolytic enzyme, DPP-4 or CD26 (EC 3.4.14.5) is expressed in all cell types of major organs [1]. The expression of DPP-4 is elevated in type 2 diabetic condition, which is responsible for the rapid degradation of incretin peptides like GLP-1 [2]. Inhibition of DPP-4 prevents degradation of incretins; higher endogenous incretin levels enhance glucose-induced insulin secretion [3]. Apart from this, DPP-4 inhibitors are also found to suppress diabetes-induced activation of NLRP3 inflammasome that induces metabolic inflammation and insulin resistance [4,5]. Thus, DPP-4 inhibitors are promising candidates for treating type 2 diabetes.

GLP-1 is a crucial incretin hormone secreted by L-cells of the distal ileum and colon. It stimulates insulin secretion in pancreatic β-cells in response to glucose [6] and, thus, plays a regulatory effect on gut motility, insulin sensitivity, controls appetite, and weight. Apart from these multiple activities, GLP-1 also exerts anti-inflammatory action [7]. The secreted GLP-1 hormone is short-lived due to its rapid degradation by the proteolytic action of DPP-4. In patients with type 2 diabetes, GLP-1 levels get impaired [8], and its effect is decreased [9].

Horsegram (*Macrotyloma uniflorum*), is a legume crop, primarily cultivated in the dry areas of Australia, Burma, India, and Sri Lanka [10]. It is reported to contain a high nutritive value with protein (23%), carbohydrates (60–70%), and fat (1%) [11]. In recent years, the potential of horsegram in diabetes has been investigated, and Myr, a major flavonol in horsegram seed coat, is reported to play a significant role in its management [12]. However, it is not clear if the bioactivity is potent when the whole horsegram (containing Myr) with proteins are consumed.

Male Wistar rats, fed with a high-fat diet and treated with a low dose of STZ (30–40 mg/ kg body weight) is reported to be a reliable model for human type 2 diabetes symptoms, which include peripheral insulin resistance, increased plasma interleukin-6 (IL-6) and hyperglycemia [13,14]. This model, reported to mimic the development of type 2 diabetes and its metabolic features, has been used by various researchers to not only investigate mechanisms involved but also evaluate potential therapies [13,14].

In the present study, the effect of Myr and HP on DPP-4 activity and its subsequent effect on GLP-1 and insulin levels in the diabetic male Wistar rat model is reported. The study also investigates the consequential impact of Myr and HP treatment on NLRP3 inflammasome and oxidative stress markers that can aggravate diabetic conditions.

## Materials and methods

### Materials

Horsegram seeds were purchased from the local market in Mysuru, India. Myr (98% purity) was commercially purchased from Sisco Research Laboratories Pvt. Ltd. Maharashtra, India. Okamet-500 tablets, purchased from a local pharmacy in Mysuru, India, were used as a source of metformin (MTF). STZ (S-0130), sodium deoxycholate, phosphatase inhibitor cocktail 2 (P-5726), phosphatase inhibitor cocktail 3 (P-0044), protease inhibitor cocktail (P-8340), phenylmethylsulfonyl fluoride (PMSF), sodium citrate, gly-pro p-nitroanilide (G-0513), recombinant DPP-4 (human) (cat no. 317639), and 4-nitroaniline were procured from Sigma-Aldrich Co, St. Louis, MO, USA. Verso cDNA Synthesis Kit was purchased from Thermo Fisher Scientific India Private Ltd, Mumbai, India. ELISA kits for cytokines IL-1β were procured from Peprotech, USA. Rat insulin ELISA kit (ERINS) was purchased from Invitrogen Bioservices, India (Pvt.) Ltd., Bengaluru, India. Antibody GLP-1 (ab23468) was purchased from Abcam, India. Antibody NLRP3 (NBP2-12446) was purchased from Novus Biologicals, India. Clarity western ECL substrate, SYBR Green qPCR mix was purchased from Bio-Rad Laboratories. Inc, USA. Primers were synthesized by Sigma-Aldrich Chemical (Pvt.) Limited, Bengaluru, India. Glucocard 01 Sensor (blood glucose strips) was procured from ARKRAY Healthcare Pvt. Ltd. Mumbai, India. PVDF membrane was purchased from Pall Life Sciences, India. All other chemicals and reagents used were of analytical grade.

### Extraction of lectin-rich protein fraction from horsegram

The horsegram seed protein fraction, rich in lectin, was isolated using a modified method of Roopashree et al., (2006) [15]. Proteins from dehulled and defatted horsegram flour were extracted using 0.1 M phosphate buffer, pH 6, at 4°C for 6 h (under stirring at 100 rpm). The resultant slurry was centrifuged at 9000 x *g* (Sorvall R 6+ centrifuge, Thermo Fisher Scientific, 96 Germany) for 40 min. The recovered supernatant was saturated with ammonium sulfate (0–40%) and left overnight at 4°C. The next day, the precipitated protein was separated by centrifugation (9000 x g for 40 min) and discarded. The supernatant was again saturated with (40–60%) ammonium sulfate and kept for 24 h at 4°C. Precipitated protein was pelleted by centrifugation and supernatant removed. The protein pellet obtained was redissolved, dialyzed extensively against water, and lyophilized. The removal of bound phenolic compounds was confirmed by HPLC. The protein content of the lyophilized pellet was analyzed for haemagglutination activity to be 1024 HAU/ mg protein and used for the animal experiment as HP. The activity was comparable to commercial lectin from horse gram. The protein content was 0.91 mg/ mg.

### Experimental design of the animal study

In the current study, male Wistar rats were used. They were bred in the animal house facility of the institute (CSIR-CFTRI) at Mysuru, India. Guidelines of the Committee for Control and Supervision of Experiments on Animals (CPCSEA), Government of India, were followed for animal care and handling. The protocols followed in the current study were specifically approved by the Institutional Animal Ethical Committee of CSIR-CFTRI, Mysuru (IAEC No. CFT/IAEC/49/2016). Animals, in polycarbonate cages (Vishnu Traders, Roorkee, Uttarakhand, India) had free access to water and feed. Three rats were housed per cage to ensure healthy development, social interactions, and well-being [16]. Animals were housed at 26 ± 2°C, under a 12/12 h light/ dark cycle, and relative humidity of 60–70%. Four-week-old male Wistar rats (n = 36), weighing around 43.6 ± 3 g, were weight-matched and acclimatized for a week, and kept on *ad libitum* semi-synthetic diet (Sai DurgaFeeds, Bengaluru, India). Required measures were taken to minimize the pain and discomfort to animals during the period of the experiment.

Method of Qian et al. (2015) [17] was followed to induce type 2 diabetes with slight modification. Initially, animals (n = 30) were given a high-fat diet (HFD) (AIN 73) (35% carbohydrates, 20% protein, and 35% fat) for four weeks. A set of rats, which received a normal fat diet (NFD) (AIN 73) (63% carbohydrates, 20% protein, and 7% fat) served as control (n = 6). The rats fed with HDF were administered with a single low dose of STZ (Sigma-Aldrich Co, St. Louis, MO, USA) (32 mg/kg body wt, dissolved in 0.1 M sodium citrate buffer at pH 4.4) intraperitoneally, whereas, control rats received an equivalent volume of citrate buffer. Food intake, weight gain, and blood glucose levels were monitored throughout the experiment. After five days of STZ administration, the blood glucose level of each rat was analyzed. Rats were showing blood glucose levels higher than 200 mg/dL were considered as diabetic and selected for further experiments. The animals were divided into six groups containing 10 rats each and administered with individual treatment molecules, once per day from the 7^th^ day onwards, as an aqueous solution of 500 μL, by oral gavaging, for four weeks.

The groups are as follows:

Group 1: NFD–Control

Group 2: HFD + STZ–DC,

Group 3: HFD + STZ + Myr (Sisco Research Laboratories Pvt. Ltd. Maharashtra, India) (Myr—20 mg/kg body wt),

Group 4: HFD + STZ + HP (HP—100 mg/kg body wt),

Group 5: HFD + STZ + mixture of Myr+HP (in the ratio of 1:5; 500 μL oral gavaging with 20 mg/kg body wt of Myr and 100 mg/kg body wt of HP)

Group 6: HFD + STZ + MTF (Okamet 500) (MTF—20 mg/kg body wt)

The respective control and diabetic control groups received an equivalent volume of saline. After four weeks of treatment, rats fasted overnight, and euthanasia was carried out by anesthetizing using CO2 followed by cervical dislocation. Blood samples were collected with 3.8% sodium citrate in the ratio of 1:9 and centrifuged after 1 h of incubation at 4°C. The liver, muscle, and intestine tissues were collected for further biochemical analysis. White adipose (perivisceral) tissue was collected by making incisions to open the abdominal cavity and collected by scraping. Plasma and all the tissues were stored at -80°C until further use.

### Estimation of blood glucose level

A small drop of blood was collected from each rat by tail snipping, and glucose levels were analyzed using glucocard glucometer (ARKRAY Healthcare Pvt. Ltd. Mumbai, India).

### Estimation of plasma insulin level

Insulin levels in plasma were analyzed using a specific ELISA kit [Rat insulin ELISA kit (ERINS), Invitrogen Bioservices India Pvt. Ltd., Bengaluru, India] as per the manufacturer’s instructions.

### *In vitro* and *in vivo* analysis of DPP-4 (CD26) activity

Initially, commercially purchased human DPP-4 enzyme (Sigma-Aldrich Co, St. Louis, MO, USA) was used to analyze the *in-vitro* effect of myricetin, HP, and Myr+HP on DPP-4 activity and to establish a standard curve.

DPP-4 activity was analyzed according to the method of Kreisel et al., (1982) [18]. Briefly, the human DPP-4 enzyme was diluted with 0.1 M Tris-HCl buffer (pH 7.5). Different concentrations of Myr (Sisco Research Laboratories Pvt. Ltd. Maharashtra, India), HP and Myr+HP (in an aliquot of 50 μL) were incubated with 10 μL of enzyme in a 96 well plate for 30 min at 37°C, and the reaction mixture made up to 0.2 mL with 0.1 M Tris-HCl buffer (pH 7.5). 100 μL of 1 mM gly-pro p-nitroanilide was added to the above and incubated for 15 min at 37°C. The enzyme activity was determined by the release of 4-nitroaniline, which was read at the absorbance of 405 nm. One unit corresponds to the hydrolysis of 1 μmol of substrate per minute per mL of protein under assay conditions.

In the *in vivo* experiment, DPP-4 enzyme activity was analyzed in liver and muscle homogenates, intestinal scrapings, and blood plasma of HFD+ STZ induced DC and treated animals. The liver and muscle tissue samples were homogenized in cell lysis buffer (10%), centrifuged, and the supernatant was taken for the analysis. The washed intestine was everted, and the subcellular mucosal surface was scraped using glass microscope slides. The scrapings were homogenized with cell lysis buffer. This intestinal homogenate was used for the analysis of DPP-4 activity. The collected blood samples with 3.8% sodium citrate in the ratio of 1:9 were centrifuged after 1 h of incubation at 4°C to separate the plasma for analysis. The samples were incubated with the mixture containing 0.1 M tris-HCl buffer (pH 8) and 1 mM gly-pro p-nitroanilide as the substrate in a reaction volume of 0.2 mL for 15 min 37°C. The enzyme activity was determined by the release of 4-nitroaniline, which was read at the absorbance of 405 nm. One unit corresponds to the hydrolysis of 1 μmol of substrate per minute per mg of protein under assay conditions.

### Protein estimation

The protein content in samples was estimated according to Lowry et al., (1951) [19].

### Estimation of antioxidant enzymes activity

Antioxidant enzymes activities (catalase, superoxide dismutase (SOD), glutathione reductase (GR), glutathione peroxidase (GPx), and glutathione transferase (GT)) were measured in liver and muscle tissues. Tissues (100 mg) were homogenized in 1 mL of 0.1M phosphate buffer, pH 7.0. Supernatants were used to assess the activities of catalase [20], SOD [21], GR [22], GPx [23], and GT [24]. Oxidative stress markers were estimated by analyzing the levels of lipid peroxidation and protein carbonyl contents. Lipid peroxides (LPO) was analyzed in liver and muscle tissues using the method of Buege and Aust(1978) [25] involving thiobarbituric acid reaction (TBA) with slight modification. Malondialdehyde (MDA) concentration, the product of lipid peroxidation, was analyzed spectroscopically to estimate the LPO level. The liver and muscle were homogenized in 0.74% KCl buffer, and an aliquot from each supernatant (200 μL) was taken to measure MDA content. Results were expressed in terms of MDA per mg of protein. Protein carbonyl content was analyzed in plasma and liver homogenate as per the method described by Mesquita et al., [26]. An equal amount of protein (3.2 mg) was taken from experimental and control samples in phosphate buffer. The final volume of the protein in phosphate buffer was adjusted to 40 μL. The sample was then incubated at ambient temperature for 10 min with 40 μL of DNPH (10 mM in 0.5 M H_3_PO_4_), and 20 μL of NaOH (6 M) was added, and incubation was continued for ten more min at ambient temperature. The absorbance was measured at 450 nm against reagent blank. Protein carbonyl content of the sample was calculated using the molar extinction coefficient of 22,000 M^-1^ cm^-1^.

### RNA extraction and gene expression analysis

Gene expression levels of CD26, PYCARD, and caspase-1 were analyzed in liver and muscle tissue. Total RNA was extracted from liver and muscle tissues using TRIzol^TM^ reagent as per the manufacturer’s instructions. The isolated RNA was transcribed into cDNA using a verso cDNA synthesis kit (Thermo Fisher Scientific India Private Ltd, Mumbai, India) according to kit instructions. Gene expressions of CD 26, PYCARD, and caspase 1 were assessed using Bio-Rad real-time PCR system (Bio-Rad, USA). The relative expression levels of the genes were normalized to GAPDH, a housekeeping gene, according to the ΔΔCt method. The sequences, 5'-CTACTTGTGTGACGTGGCCT-3' and 5'-TAGTCGCAGATCGCCATCAC-3' ware used as forward and reverse primers for CD26, respectively. For the PYCARD gene, sequence 5'-AGAGACCCCCAACCAAAACA-3' was used as the forward primer, and 5'-CAAGTAGGGCTGTGTTTGCC-3' was used as a reverse primer.5’-AACACCCACTCGTACACGTC-3’ and 5’-TGAGGTCAACATCAGCTCCG-3’ ware used as forward and reverse primers for caspase-1, respectively. For GAPDH, 5’-GCCATCAACGACCCCTTCAT-3’ and 5’-AGATGGTGATGGGTTTCCCG-3’ were used as forward and reverse primers, respectively (supplied by Sigma-Aldrich, Bengaluru, India).

### Western blot analysis

Protein expression of GLP-1 and NLRP3 were analyzed in blood plasma and liver tissue, respectively, by the immunoblot technique. Blood samples, collected with 3.8% sodium citrate (ratio of 1:9), were centrifuged, after 1 h of incubation at 4°C, to obtain plasma. RIPA buffer, containing 50 mM Tris-HCL pH8, 0.5% sodium deoxycholate, 150 mM sodium chloride, 1% Triton X-100, 0.1% SDS, 1 mM PMSF, phosphatase inhibitor and protease inhibitor cocktail (1%), was used to prepare liver homogenate (10%). The plasma and liver homogenate samples were estimated for protein content using the method of Lowry et al. (1951) [19]. Plasma and liver samples were diluted to 1:1000 and 1:500, respectively, for western blot analysis.

Samples were subjected to SDS PAGE gel electrophoresis, according to Laemmli (1970), using 10% gels [27]. The separated protein bands were transferred to a PVDF membrane (pore size 0.2 μM, Pall Life Sciences, India); the membrane was blocked with 5% BSA to avoid non-specific binding. Excess of bovine serum albumin (BSA) was washed with TBST, and the membrane was incubated with primary antibodies against GLP-1 (Abcam, India) (1:1000 dilution) and NLRP3 (Novus Biologicals, India) (1:1000 dilution), independently, for 2 h, at ambient temperature. Transferrin and β-actin were used as loading controls for GLP-1 and NLRP3, respectively. At the end of the incubation, the membrane was washed with TBST to remove unbound antibodies and treated with the respective HRP-conjugated secondary antibody for 1 h at ambient temperature. The blots were washed with TBST to remove unbound secondary antibodies. Using enhanced chemiluminescence reagent (Clarity Western ECL substrate, Bio-Rad Laboratories.Inc., Bengaluru, India), blots were developed for protein bands and analyzed using the Syngene Gel Doc system facilitated with blue light transilluminators (G: BOX Chemi XT4).

### Estimation of IL-1β levels

Interleukin 1β (IL-1β) was estimated in plasma using a specific ELISA kit (Peprotech, USA) according to the manufacturer's instructions.

### Statistical analysis

The rats, used in the study, were checked for general health, and no rat was eliminated from the study. Results are presented as mean ± standard deviation. A comparison among the groups was performed using ANOVA (nonparametric). Post-ANOVA, multiple comparisons between the mean of each group with the mean of every other group, were performed by Tukey's test. Differences were considered significant only if their adjusted P-value (P_adj_) was < 0.05, with a 95% confidence interval. All statistical analyses were performed and graphs generated using Graph Pad Prism version 6.02 for Windows (Graph Pad Software, La Jolla, CA, USA; www.graphpad.com)

## Results

### Analysis of the *in-vitro* effect of Myr and HP on DPP-4 enzyme activity

**Fig 1:**
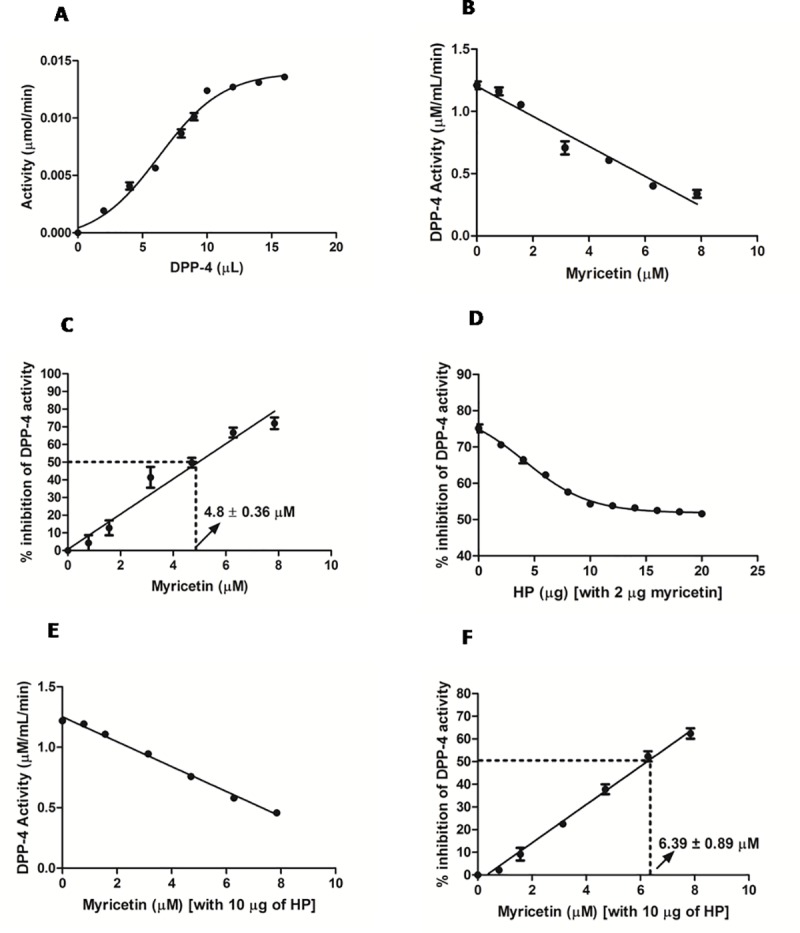
*In-vitro* effects of myricetin and HP on DPP-4 activity. A. Standard curve for DPP-4 activity. B-C. Inhibition effect of Myr on DPP-4 activity. Myr inhibited DPP-4 activity with an IC_50_ value of 4.8 ± 0.36 μM. D. Inhibitory effect of Myr, in presence of different ratios of HP on DPP-4 activity. The DPP-4 inhibitory activity of Myr was reduced in the presence of HP in the ratio of 1:5. E-F. Effect of HP (10 μg) on the DPP-4 inhibitory activity of Myr. In the presence of HP, Myr exhibited reduced DPP-4 inhibition activity, and its IC_50_ value shifted to 6.39 ± 0.89 μM. Data are mean of triplicate values.

The activity of the commercially purchased DPP-4 enzyme was determined to be 1.028 ±0.124 μmol/mL/min. **Fig 1A** shows the standard curve of DPP-4 enzyme activity; 10 μL of the enzyme showed maximum activity. Further higher concentrations of the enzyme did not reveal any further increase in the activity. Hence further analysis of DPP-4 activity with other test molecules was carried out using 10 μL of the enzyme. The activity of the commercial DPP-4 enzyme was found to be 1.028 ± 0.124 μmol/ml/min. *In-vitro* effects of Myr, HP, and Myr + HP on DPP-4 enzyme activity were analyzed. Myr inhibited DPP-4 activity (**Fig 1B**) with an IC50 value of 4.8 ± 0.36 μM (**Fig 1C**), while, HP did not have any effect. DPP-4 inhibition capacity of Myr was analyzed in the presence of different concentration of HP, where Myr (2 μg) and HP (10 μg) in the ratio of 1:5 showed lesser inhibition activity (**Fig 1D**). Hence, different molar concentration of Myr along with 10 μg of HP were analyzed for enzyme inhibition activity. **Fig 1E** shows the enzyme inhibitory activity of Myr in the presence of HP. Myr, in the presence of HP, had reduced inhibition capacity (40%) and its IC50 value shifted to 6.39 ±0.89 (**Fig 1F**).

### Animal food consumption

Four-week rats were acclimatized for a week on *ad libitum-*semisynthetic diet. During the period, the food consumption of the rats was 35.7 ± 63 g/rat/week. After 5^th^-week, animals were divided into NFD and HFD groups. Table 1 shows the food consumption of the animals during the experimental period. T-test was conducted to analyze the difference in food consumption of the rats fed with NFD and HFD. No significant difference was observed between NFD and HFD in food intake up to 9^th^ week. After the administration of STZ in the 10^th^ week, there was no significant difference observed in the food intake between NFD and HFD groups. During treatment with test molecules, there was no significant difference observed at 11^th^ and 12^th^ week among control, DC, and treatment groups. In the 13^th^ week, there was a significant (P<0.05) reduction in the food consumption of DC (57%), Myr (60%), HP (57%), Myr+HP (50%), and MTF (55%) groups compared to control. Similarly, in the 14^th^ week also, there was a significant (P<0.05) reduction in food intake of DC (59%), Myr (63%), HP (58%), Myr+HP (54%), and MTF (64%) groups compared to control.

**Table 1:** Growth parameters of experimental rats.

	Weight gain/ 10 weeks (g)	Food intake/ 10 weeks (g)	Food efficiency ratio (FER)	Liver weight (g)	Adipose tissue weight (g)
**control**	172.2 ± 14.1	852.2 ± 50.6	0.20 ± 0.01	5.67 ±0.45	2.80 ±0.36
**DC**	124.7 ± 20.8*	646.7 ± 61.8*	0.19 ± 0.02 ^NS^	5.83 ±0.25^NS^	1.70 ±0.62*
**Myr**	139.3 ± 15.7*	639.6 ± 85.2*	0.22 ± 0.03 ^NS^	5.33 ±0.50 ^NS^	1.33 ±0.32*
**HP**	112.8 ± 15.0*	638.6 ± 59.7*	0.18 ± 0.02 ^NS^	5.87 ±0.31 ^NS^	2.20 ±0.10 ^NS^
**Myr+HP**	134.1 ± 18.0*	665.4 ± 28.4*	0.20 ± 0.01 ^NS^	5.57 ±0.64 ^NS^	2.00 ±0.10 ^NS^
**MTF**	141.3 ± 8.3^NS^	635 ± 65.2*	0.22 ± 0.02 ^NS^	5.60 ±0.20 ^NS^	1.07 ±0.35*

Values are represented as mean ± SD (*n* = 6).

*p< 0.05 when compared to control and

NS–not significant when compared to control.

### Animal body weight

**Table 1** represents the body weights of rats during the experimental period. 4-week old Wistar male rats weighed an average weight of 43.6 ±3 g. After a week of acclimatization, 5-week old rats (58.7 ±4.8 g) were grouped into NFD and HFD groups. The growth parameters of experimental rats are given in **Table 1**. The food and weight gain of the DC group was lower than the control. However, Food efficiency ratio (FER) were not significantly different among the groups.

T-test was conducted to analyze the changes developed in body weights for the rats fed with NFD and HFD. In the 6^th^ week, there were no significant changes in the bodyweight of HFD rats compared to the NFD group. A significant (P<0.05) increase (9.3%) in the bodyweight of HDF was observed compared to NFD in the 7^th^ week. In the 8^th^ and 9^th^ weeks, the bodyweights of HFD was significantly (P<0.05) high (11.3% and 9.6%, respectively) compared to the NFD fed group. A week after the administration of STZ to HFD fed group, the body weights were significantly (P<0.05) reduced (16.5%) compared to non-STZ treated NFD fed rats. During four weeks of treatment with test molecules, body weights were monitored. The changes in the bodyweights among groups were analyzed by one way ANOVA Tukey's test. There were no significant difference in body weights among the groups until the 13^th^ week. In the 14^th^ week, there was a significant (P<0.05) loss of body weight in the DC group (24.6%), HP (24.5%), and Myr+HP (15.3%) compared to control group.

### Analysis of blood glucose levels

Four-week-old rats were acclimatized for a week, and the blood glucose levels were found to be 104.6±4.1 mg/dL (**Table 2**). After grouping the rats as NFD and HFD fed rats, in 6^th^ week, there was no significant difference in blood glucose level between NFD and HFD. In the 7^th^ week, a significant (P<0.05) high (10%) blood glucose level was observed in HFD fed rats compared to NFD. In the 8^th^ week, the HFD group showed an 18% increase (P<0.05) in the blood glucose level compared to NFD. The blood glucose level of HFD elevated by 26% in the 9^th^ week compared to NFD (Table 3). After the administration of STZ to the HFD group at the 10^th^ week, the blood glucose levels showed above 200 mg/dL, i.e., 444 ± 29.5 mg/dL. After the confirmation of onset of diabetes by elevated blood glucose level (>200 mg/dL), rats were divided into groups and administered with Myr, HP, Myr+HP, and MTF, individually, for four weeks. The blood glucose levels were monitored and shown in **Table 2**. The DC rats, without treatment, exhibited significantly (P<0.05) high blood glucose levels compared to the control group during the period. Myr treated groups showed significant (P<0.05) decrease in the blood glucose level by 12%, 9%, 11%, and 28% during the 11^th^,12^th^, 13^th^ and 14^th^ weeks, respectively, compared to DC. HP had no significant effect on elevated blood glucose level at 11^th^, 12^th^, and 13^th^ week, however, the group exhibited reduced (13%) elevated blood glucose level in the 14^th^ week of treatment compared to DC. There was a significant (P<0.05) reduction in the elevated blood glucose level in the group treated with Myr+HP in 11^th^, 12^th^, 13^th^ and 14^th^ week by 20%, 27%, 27%, and 11%, respectively, compared to DC. MTF treatment was significantly (P<0.05) efficient in the reduction of elevated blood glucose level by 20%, 23%, 27%, and 33% during the treatment period of 11^th^,12^th^, 13^th^, and 14^th^ weeks, respectively, compared to untreated DC rats. At the end of the 14^th^ week, the rats were fasted, and the fasting blood glucose levels were recorded. The DC rats had significantly (P<0.05) elevated fasting blood glucose levels compared to control rats. Myr, HP, Myr+HP, and MTF treated groups exhibited significant (P<0.05) reduction in the elevated fasting blood glucose level by 38%, 19%,30, and 59%, respectively, compared to DC.

**Table 2:** Blood glucose levels of experimental rats (mg/dL).

**Weeks**	**5^th^ week**		**6^th^ week**	**7^th^ week**	**8^th^ week**	**9^th^ week**		**10^th^ week**		**11^th^week**	**12^th^week**	**13^th^week**	**14^th^ week**	**14^th^ week (fasting)**
Blood glucose level (mg/dL)	104.6±4.1	*NFD*	100.3±6.6	109±4	106.3±3.7	106.3±9.2		106 ± 1.5	**Control**	103 ± 13.8	87 ±2.2	87 ± 2.2	92±9.3	65 ± 1.0
		*HFD*	117±4	122±4^a^	130.6±4.5^a^	144.6±7.6^a^	STZ administration	444 ± 29.5*	**DC**	425 ± 35.1*	382 ± 3.0*	370 ± 40.2*	380±22.4*	353 ± 8.1*
									**Myr**	370 ± 17.0^#^	347 ± 25.3^#^	328 ± 37.5^#^	270± 5.8^#^	216 ±48.1^#^
									**HP**	455 ± 45.5^NS^	369 ± 32.2^NS^	351 ± 81.2^NS^	328±12.4^NS^	284 ± 21.6^#^
									**Myr+HP**	336 ± 8.8^#^	277 ± 28.3^#^	268 ± 22.9^#^	336±14.6^NS^	245 ± 21.0^#^
									**MFT**	337 ± 18.1^#^	294 ± 26^#^	270 ± 11.4^#^	254±19.1^#^	143 ± 5.0^#^

Values are represented as mean ± SD (*n* = 6).

^a^*P*<0.05 when compared to NFD rats.

*P< 0.05 when compared to control

^#^*P*< 0.05 when compared to DC and

NS–not significant when compared to DC group

**Table 3:** DPP-4 activity affected by the presence of Myr and HP (μmol/mg/min).

	**Liver**	**Muscle**	**Plasma**	**Intestine**
**Control**	6.05 ± 0.40	1.20 ± 0.007	0.93 ±0.07	38.06 ± 2.58
**DC**	10.35 ± 1.30*	2.11 ± 0.04*	1.35 ±0.07*	50.86 ± 4.94*
**Myr**	5.08 ± 0.36^#^	1.50 ± 0.17^#^	0.65 ±0.05^#^	28.38 ± 2.32^#^
**HP**	9.86 ± 0.83^NS^	1.83 ± 0.03^NS^	0.81 ±0.08^#^	43.46 ± 2.50^NS^
**Myr+HP**	7.80 ± 0.38^#^	1.66 ± 0.01^#^	1.22 ±0.04^NS^	49.01 ± 1.62^NS^
**MTF**	8.11 ± 0.59^#^	1.78 ± 0.10^NS^	1.27 ±0.09^NS^	44.32 ± 3.83^NS^

Values are represented as mean ± SD (*n* = 6).

*P< 0.05 when compared to control

^#^*P*< 0.05 when compared to DC and

NS–not significant when compared to DC group.

### Estimation of DPP-4 activity

The enzyme activity of DPP-4 was estimated in the liver, muscle, plasma, and intestinal scrapings of experimental rats (**Table 3**). DC rats exhibited significantly (P<0.05) increased DPP-4 activity in liver (41%), muscle (43%), plasma (31%), and intestine (25%), respectively, compared to control. In liver homogenate, Myr treated group exhibited a significant (P<0.05) reduction (50%) in DPP-4 activity compared to the DC group followed by Myr+HP (24%) and MTF (21%). There was no significant decrease in liver DPP-4 activity in the group fed with HP (P<0.05 when compared with DC). There was a notable reduction in muscle DPP-4 activity in the groups treated with Myr (28%) and Myr+HP (21%) (P<0.05 when compared with DC). The groups administered with HP and MTF didn’t show any significant reduction in muscle DPP-4 activity (P<0.05). In plasma, the DPP-4 activity was significantly reduced in the groups treated with Myr (51%) and HP (40%) (P<0.05 when compared with DC). There was no notable lowering of plasma DPP-4 activity in the groups fed with Myr+HP and MTF compared with DC rats (P<0.05). The intestinal DPP-4 activity was decreased significantly in the group treated with Myr (44%); other treatment groups did not exhibit any notable reduction in the intestinal DPP-4 activity (P<0.05 when compared with DC). As can be seen, Myr treatment exhibited the best effect in lowering DPP-4 activity to near control in all the tissues as well as plasma (**Table 3**).

### Analysis of DPP-4 gene expression

**Fig 2:**
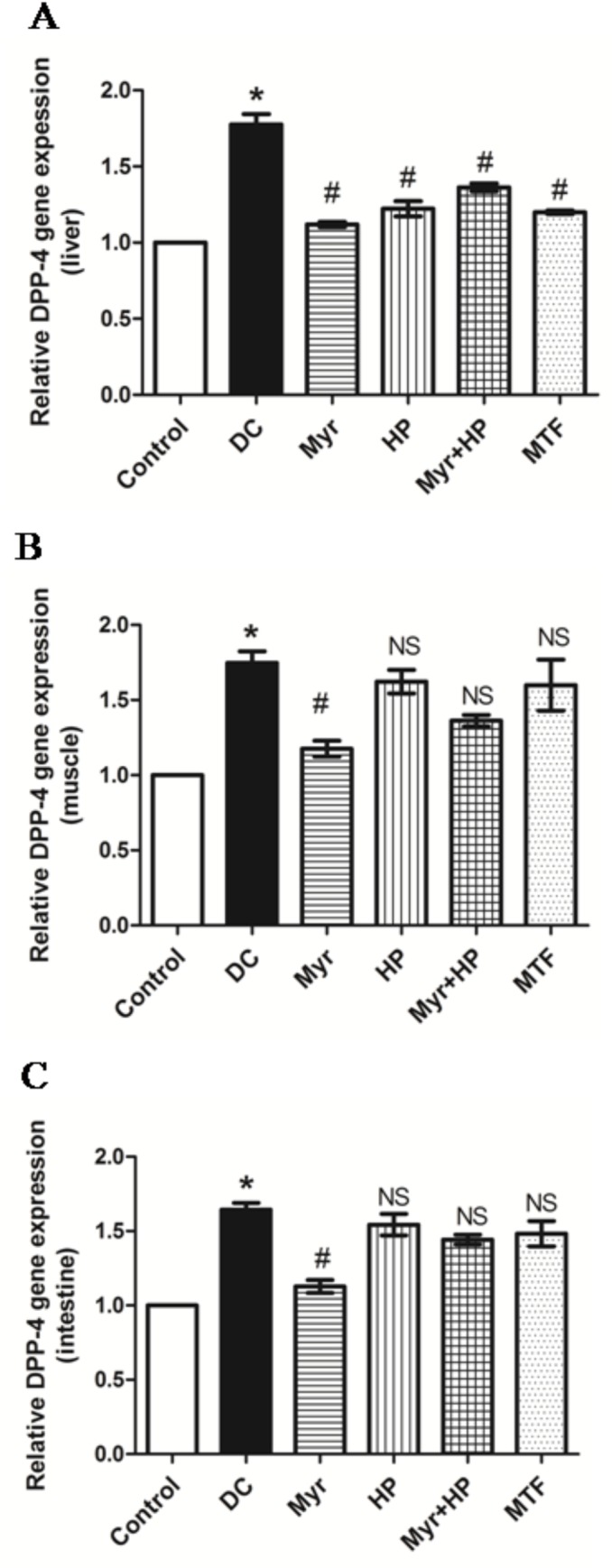
Evaluation of DPP-4 gene expression in different tissues. (A) Relative gene expression of DPP-4 in the liver. All the test molecules significantly suppressed DPP-4 gene expression in the liver compared to DC. (B) Relative gene expression of DPP-4 in muscle. Myr treatment significantly reduced DPP-4 gene expression in muscle, while HP, Myr+HP, and MTF treatment had no significant effect. (C) Relative gene expression of DPP-4 in the intestine. Myr was effective in the reduction of DPP-4 gene expression in intestinal tissue compared to DC rats, while HP, Myr+HP, and MTF treatment had no significant effects. Data are mean ± SD (*n* = 6). *P< 0.05 when compared to control. ^#^*P*< 0.05 when compared to DC and NS–not significant when compared to the DC group.

Gene expression of DPP-4 was analyzed in the liver, muscle, and intestinal tissues by RT-PCR (**Fig 2**). DPP-4 gene expression was significantly increased (P<0.05) in the DC group in all tissues studied—liver (43%), muscle (42%), and intestine (37%) compared to control (**Fig 2**). Myr treatment showed better reduction (37%) of DPP-4 gene expression in liver tissue followed by MTF (32%), HP (31%), and Myr+HP (23%) (P<0.05, when compared with DC) (**Fig 2A**). Myr alone was found to be 17% better in the suppression of the DPP-4 gene expression compared to Myr+HP fed group. In muscle and intestinal tissues, Myr administered group exhibited suppressed DPP-4 gene expression by 42% and 28%, respectively, (P<0.05, when compared with DC), while, in other treated groups exhibited no significant effect (**Fig 2B** and **Fig 2C**).

### GLP-1 protein expression and plasma insulin levels

**Fig 3:**
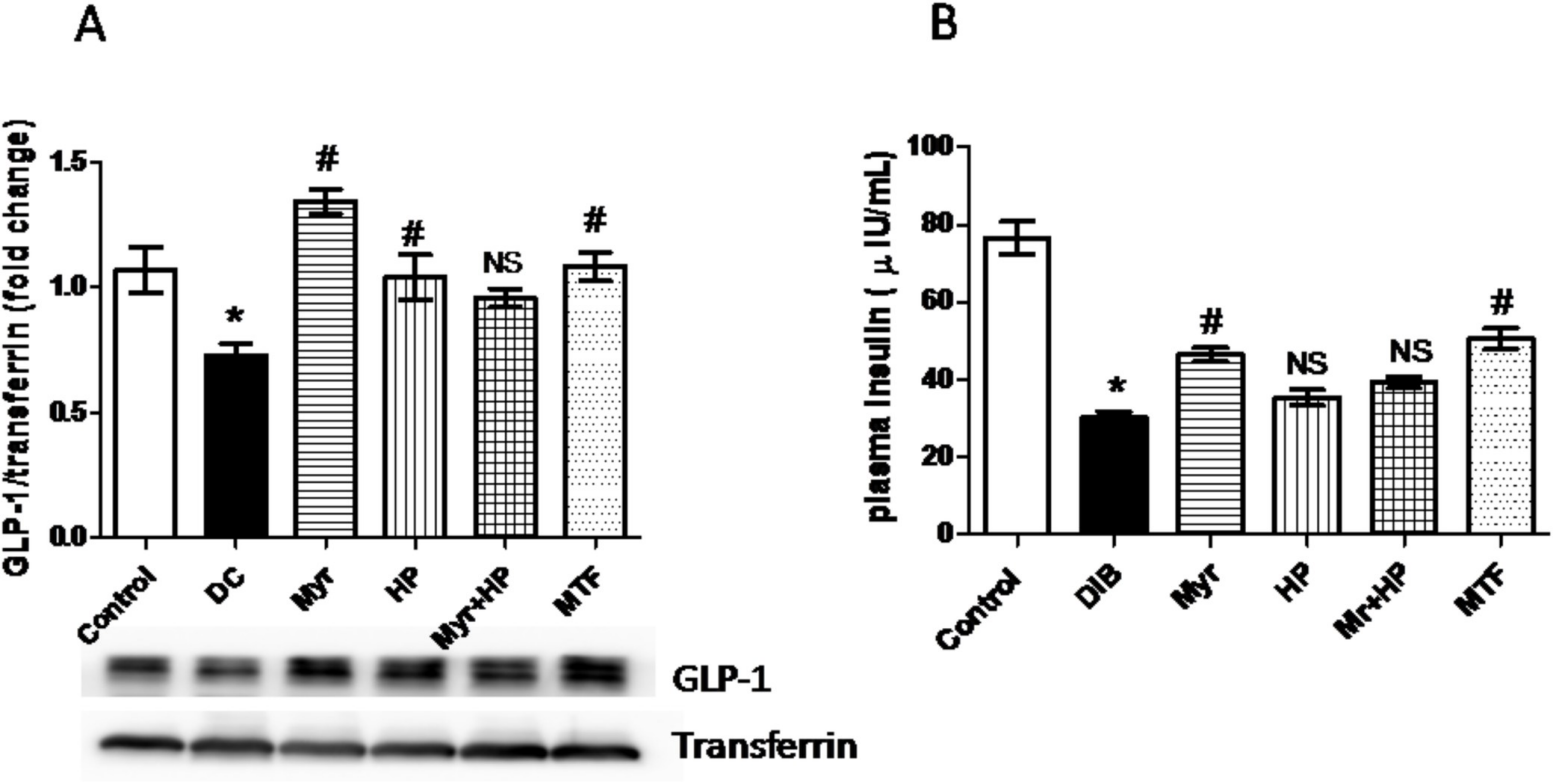
Estimation of circulating GLP-1 and plasma insulin levels. (A) Plasma GLP-1 levels estimated by immune-blot technique. DC rats showed reduced expression of GLP-1 protein compared to control. Myr, HP, and MTF were effective in elevating GLP-1 levels, while Myr+HP had no significant effect. (B) Plasma insulin levels. Compared to control, DC rats exhibited lower plasma insulin levels. Myr and MTF were efficient in recovering plasma insulin levels compared to DC. HP and Myr+HP had no significant effect on plasma insulin levels. Data are mean ± SD (*n* = 6). *P< 0.05 when compared to control. ^#^*P*< 0.05 when compared to DC and NS–not significant when compared to the DC group.

Circulating GLP-1 protein was quantified in plasma by immunoblot technique and compared between the groups. As can be seen in **Fig 3A**, DC exhibited a significant (P<0.05) decrease (32%) in the GLP-1 protein level compared to control. Myr treated group revealed increased GLP-1 levels in plasma (46%) followed by MTF (33%) and HP (30%) fed groups (P<0.05 when compared to the diabetic group). There was no notable increase in plasma GLP-1 level in the group administered with Myr+HP. The possible effect of DPP-4 inhibition in enhancing the GLP-1 activity needs to be further experimentally scrutinized.

**Fig 3B** represents the plasma insulin levels in the experimental rats. DC rats exhibited a significantly (P<0.05) lower level (60.6%) of plasma insulin compared to control. There was a significant (P<0.05) enhancement in plasma insulin levels in the groups treated with Myr and MTF (78.7% and 87.7%, respectively), compared to DC. HP and Myr+HP had no significant effect on plasma insulin levels (**Fig 3B**).

### Measurement of antioxidant enzyme activities and oxidation stress markers

The activities of antioxidant enzymes—catalase, SOD, GR, GPx, and GT were measured in the liver (**Table 4**). In DC rats, a significant (P<0.05) reduction in all the enzyme activities of catalase (40%), SOD (55%), GR (41%), GPx (35%), and GT (22%) were observed, compared to control. There was a significant (P<0.05) recovery of calatase enzyme activity in the groups treated with Myr (20%), HP (22%), Myr+HP (25%), and MTF (27%) (P<0.05, when compared with DC).

**Table 4:** Antioxidant enzyme activities in liver.

	Catalase (μmol/min/mg protein)	SOD (Unit/min/mg protein)	GR (nmol/min/mg protein)	GPx (nmol/min/mg protein)	GT (nmol/min/mg protein)
**Control**	149.37 ± 4.04	3.20 ± 0.20	95.01 ± 8.9	233.32 ± 7.4	0.36 ± 0.009
**DC**	89.41 ± 11.89*	1.44 ± 0.09*	55.95 ± 4.5*	151.51 ± 9.7*	0.28 ± 0.038^NS^
**Myr**	112.98 ± 3.92^#^	1.98 ± 0.05^#^	123.38 ± 6.4^#^	300.35 ± 7.78^#^	0.55 ± 0.069^#^
**HP**	115 ± 7.81^#^	2.30 ± 0.16^#^	117.01 ± 5.3^#^	248.46 ± 4.8^#^	0.50 ± 0.082^#^
Myr+HP	120.01 ± 7.5^#^	2.21 ± 0.17^#^	144.93 ± 9.5^#^	301.97 ± 15.4^#^	0.38 ± 0.082^NS^
MTF	123.16 ± 12.21^#^	2.13 ± 0.21^#^	126.73 ± 8.2^#^	307.97 ± 10.0^#^	0.51 ± 0.091^#^

Values are represented as mean ± SD (*n* = 6).

*P< 0.05 when compared to control

^#^*P*< 0.05 when compared to DC and

NS–not significant when compared to DC group.

The observed activity of SOD was elevated on the administration of Myr (27%), HP (37%), Myr+HP (34%), and MTF (32%) (P<0.05, when compared to DC). Similarly, the administration of Myr, HP, Myr+HP, and MTF increased liver GR activity by 54%, 52%, 61%, and 55%, respectively, (P<0.05, when compared to DC). Liver GPx activity was also found to be enhanced by 49%, 39%, 49%, and 50% in the groups treated with Myr, HP, Myr+HP, and MTF, respectively (P<0.05, when compared with DC). In the same manner, GT activity was significantly (P<0.05) enhanced in the liver by 49%, 44%, 26%, and 45% in the groups fed with Myr, HP, Myr+HP, and MTF, respectively, compared to DC rats.

As a marker of oxidative stress, the level of lipid peroxidation (LPO) was estimated in the liver, and the level of protein carbonyls was estimated in the liver and plasma (**Table 5**). A marked elevation (33%) of liver LPO was observed in the DC group (P<0.05, when compared with control). Myr, Myr+HP, and MTF treated groups revealed a significant reduction in lipid peroxidation by 52%, 49% and 56%, respectively, (P<0.05 when compared to DC) in the liver. No significant (P<0.05) effect was noticed in HP administered group in lowering of liver lipid peroxidation compared to other treated groups. In the analysis of protein carbonyl levels (**Table 5**), DC rats showed a significantly (P<0.05) high levels (92.1%) of liver protein carbonyls compared to control. Myr, HP, Myr+HP and MTF treatment significantly (P<0.05) reduced the liver protein carbonyl by 55.2%, 37.2%, 33.7% and 50.5%, respectively. Similarly, in comparison with the control group, DC rats exhibited significantly (P<0.05) increased (50.8%) level of plasma protein carbonyls. There was a significant (P<0.05) decrease in the plasma protein carbonyls in the groups treated with Myr (37.6%), HP (56.5%), Myr+HP (41.9%) and MTF (49.2%)

**Table 5:** Estimation of oxidative stress markers.

	Liver LPO (nmol/mg of protein)	Liver protein carbonyls (nmols/mg of protein)	Plasma protein carbonyls (nmols/mg of protein)
**Control**	7.64 ± 0.77	2.64 ± 0.23	0.63 ± 0.01
**DC**	11.51 ± 0.37*	5.08 ± 0.20*	0.95 ± 0.01*
**Myr**	5.47 ± 0.47^#^	2.28 ± 0.16^#^	0.59 ± 0.03^#^
**HP**	8.59 ± 0.14^NS^	3.19 ± 0.29^#^	0.41 ± 0.00^#^
**Myr+HP**	5.78 ± 0.29^#^	3.37 ± 0.14^#^	0.55 ± 0.03^#^
**MTF**	4.98 ± 0.54^#^	2.52 ± 0.15^#^	0.48 ± 0.03^#^

Values are represented as mean ± SD (*n* = 6).

*P< 0.05 when compared to control

^#^*P*< 0.05 when compared to DC and

NS–not significant when compared to DC group.

### Evaluation of proteins involved in NLRP3 inflammasome

**Fig 4:**
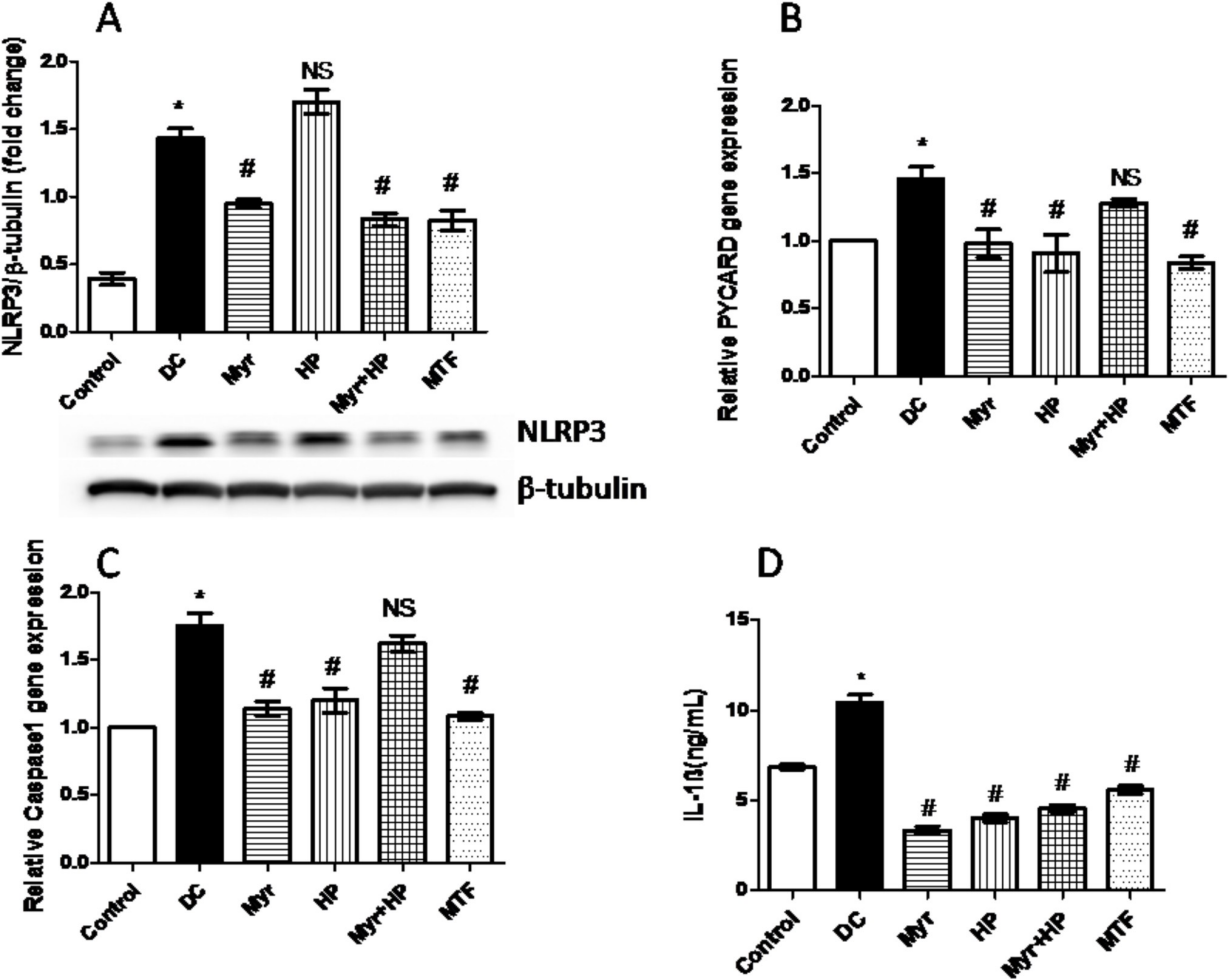
Analysis of modulation of proteins involved in the NLRP3 inflammasome. (A) Estimation of liver NLRP3 by immune-blot. Myr, HP, and MTF treatments were effective in reducing the level of NLRP3 protein. (B-C) Relative gene expression of PYCARD and Caspase-1 in the liver. Myr, HP, and MTF administration significantly reduced relative gene expressions of PYCARD and caspase-1. (D) Estimation of IL-1β by specific ELISA kit. All the treatment molecules significantly reduced the IL-1β levels compared to DC rats. Data are mean ± SD (*n* = 6). *P< 0.05 when compared to control. ^#^*P*< 0.05 when compared to DC and NS–not significant when compared to the DC group.

The level of NLRP3 protein expression was analyzed in liver tissue by immune-blot method. There was a profound increase (73%) in the expression of NLRP3 in DC rats (P<0.05, when compared with control) (**Fig 4A**). Myr, Myr+HP, and MTF treatment significantly (P<0.05) reduced the elevated NLRP3 levels by 34%, 41%, and 42%, respectively, compared to DC. The group administered with HP did not show any significant difference in the levels of NLRP3 (P<0.05, when compared to DC) (**Fig 4A**). Gene expressions of PYCARD and caspase-1 were analyzed in liver tissue using RT-PCR (**Fig 4B and 4C**). DC rats exhibited a significant (P<0.05) increase in PYCARD (31%) and caspase-1 (42%) compared to control. PYCARD gene expressions were significantly lowered in the groups treated with Myr (32%), HP (37%), and MTF (42%) (P<0.05, when compared with DC). Similarly, Myr, HP, and MTF administered groups had lowered caspase-1 gene expression by 35%, 31%, and 38%, respectively (P<0.05, when compared with DC). No significant (P<0.05) changes in PYCARD and caspase-1 gene expression were observed in Myr+HP fed group, compared to DC rats. The circulating IL-1β levels were estimated in blood plasma. DC rats exhibited elevated (51%) levels of IL-1β (P<0.05, when compared to control). All the treatment groups had effectively reduced elevated plasma IL-1β. Myr, HP, Myr+HP, and MTF treatment significantly (P<0.05) lowered plasma IL-1β by 66%, 59%, 56%, and 45%, respectively (**Fig 4D**).

## Discussion

There are well established therapeutic treatments available for the effective management of blood glucose for type 2 diabetes. The first line of treatment includes biguanides, sulfonylureas, meglitinides, thiazolidinediones, and DPP-4 inhibitors [28]. MTF, generally used as the first line of medication, belongs to the class of biguanides and brings about its effect by increasing insulin sensitivity [29]. Sulfonylureas and meglitinides are reported to increase insulin secretion from pancreatic β-cells [30]. As inhibition of DPP-4 prolongs the life of incretins (GLP-1 and GIP), which in turn induce insulin secretion, many DPP-4 inhibitors like sitagliptin and vildagliptin are emerging as powerful adjuncts in the treatment of type 2 diabetes [31]. Gliptins (including 4 FDA approved drugs) are inhibitors of DPP-4 that further prevent degradation of GLP-1 [32]. Even though these therapeutics are found to be effective in the regulation of blood glucose levels, side effects, on long term use, are of major concern.

Natural molecules like Myr, a potent DPP-4 inhibitor, can be of great value and a potential adjunct therapeutic molecule for managing type 2 diabetes. Our study reveals the anti-hyperglycemic effect of Myr and its possible mechanism through DPP-4 inhibition *in-vitro* and *in-vivo*. As evident from our results, Myr treatment reduced elevated blood glucose levels (**Table 2**). Myr was efficient in recovering intact GLP-1 peptides that could be the result of DPP-4 inhibition. Also, Myr treatment improved insulin levels (**Fig 3B**) as a probable consequence of intact GLP-1 restoration; this, in turn, could be responsible for the anti-hyperglycemic effect (**S1 Fig**). Apart from this, Myr also brought down the gene expression levels of DPP-4 (**Fig 2**), which accounts for the reduced DPP-4 activity observed. The effect of Myr in modulating DPP-4 (at the transcriptional level) needs further experimental scrutinization.

Inhibition of DPP-4 has been reported to lower inflammation and also repress inflammasome [5]. An inflammasome is a multimeric protein complex that can sense damage-associated molecular patterns (DAMPs) and control the secretion of proinflammatory cytokines, interleukin IL-1β and IL-18 [33]. It is also well established that pro-inflammatory cytokines can induce insulin resistance. Inflammasome, NLRP3, is a well-characterized complex, which gets activated in obesity-related type 2 diabetes. Structurally, NLRP3 consists of the node-like receptor (NLR), the apoptosis-associated spec-like protein containing a PYCARD (ASC) adaptor protein, and caspase-1. Activation of caspase-1 controls the cleavage and secretion of pro-IL-1β into bioactive cytokine [4]. Our study also takes into account the effect of Myr on NLRP3 inflammasome and oxidative stress markers that aggravate type 2 diabetes. Reduced levels of NLRP3 inflammasome were observed in Myr treated animals (**Fig 4A**). The observed effect could also be a parallel or independent effect of DPP-4 inhibition by Myr. As a consequential effect, PYCARD and caspase-1 expressions were reduced (**Fig 4B and 4C**), though the transcriptional effect of Myr on these genes is unknown. The reduction of the NLRP3 inflammasome by Myr is reflected in a reduction in the cytokine IL-1β (**Fig 4D**). Myr, a powerful antioxidant [12], reduced the oxidative stress markers LPO and protein carbonyls and also enhanced the antioxidant enzyme activities in our studies (**Table 5**).

Oxidative stress and inflammation are intricately connected and regulated by each other [34,35]; oxidative stress coupled with chronic inflammation can lead to insulin resistance [36]. GLP-1 peptides are reported to bring down oxidative stress levels and improve antioxidant enzyme activity in tissues [27]. Flavonoids are known to scavenge reactive oxygen species, protect and restore antioxidant enzymes [37]. Quercetin, a flavonol, when administered intraperitoneally, to STZ induced diabetic rats, is reported to improve glucose tolerance and dyslipidemia by reducing oxidative stress and protection against β-cell apoptosis [38]. As evident from this study, lowered antioxidant enzymes levels and activity and increased lipid peroxidation were observed in DC rats. However, treatment with Myr improved the antioxidant enzyme activities and lowered lipid peroxidation, irrespective of its ability to restore intact GLP-1 levels. As observed from this study, in DC rats, NLRP3 protein, PYCARD, and caspase-1 are found to be expressed at higher levels along with plasma IL-1β levels. Though Myr+HP treatment reduced the NLRP3 expression level, it had no significant effect on PYCARD and caspase-1 expression. It is interesting to note that the administration of Myr, a powerful antioxidant and also anti-inflammatory molecule [12], lowered the NLRP3 levels, which needs to be further studied in connection with insulin sensitivity in muscles and adipose tissue. The findings of the study are summarized in **S1 Fig**

Horsegram, rich in the polyphenol–myricetin and containing 23% protein [11], is generally consumed whole. HP had no significant effect on elevated blood glucose level throughout the experiment (**Table 2**). HP did not have any effect on DPP-4 activity. However, at the end of the experiment (14^th^ week), fasting blood glucose levels were significantly reduced by HP, but the mechanism still needs to be explored. In addition, when HP was given with Myr, DPP-4 inhibition was lowered considerably (**Table 3**), albeit not as much as observed with Myr alone, indicating that Myr alone was probably responsible (for the inhibition). Though HP and Myr+HP fed groups revealed significant reduction in DPP-4 gene expression, they were not consistent and less effective in comparison to Myr alone (**Fig 2**).

Though HP had slight recovering ability of GLP-1, it had no significant effect on insulin levels (**Fig 3**), also HP+Myr had no impact on recovering GLP-1 and insulin levels. This again proves that, Myr lost its modulatory effect in the presence of HP. Further, although HP had no effect on NLRP3 inflammasome, it brought down the IL-1β levels. However, in combination with Myr, PYCARD and caspase-1 gene expression reduction were not seen and reduction of IL-1β was not superior to Myr treatment (**Fig 4**). HP alone and along with Myr significantly enhanced the antioxidant enzyme activities and reduced oxidative stress markers. The mechanism is still not clear and needs further studies.

Loss in bodyweight was observed in rats treated with Myr, HP and HP+Myr. Several flavonoids are reported to induce browning of white adipose tissue and increase thermogenesis. Energy expenditure is higher when flavonoids are consumed and this could be one reason for bodyweight loss [39]. Dihydromyricetin is reported to stimulate irisin secretion through the PGC-1α pathway partially. Irisin is postulate UCP1 expression in white adipose tissue that increases energy expenditure in mice and induces white adipose tissue browning. This is also reported in case of treatment of experimental mice with quercetin and rutin [39].

Flavonoids, known for their antioxidant activities, are reported to bind to proteins [40]. When bound to proteins in the food matrix, these antioxidant molecules do not attain their maximum scavenging capacity. Further, interaction and binding to proteins, may decrease their bioavailability. Although, binding of flavonoids to proteins may be reversible or irreversible, depending on pH, temperature, protein and polyphenol concentration, the decrease in free flavonoid content results in reduced antioxidant potential [40]. Lectins are proteins that are difficult to digest in native form. They hinder digestion and absorption by binding to cells lining the digestive tract [41]. This also hinders absorption of other nutrients and over long period, may cause diarrhea and nausea and result in bodyweight loss [41]. Thus, in addition to the amount of bioactive molecule, its bioavailability and activity, it is important to consider its interactions with co-nutrients for understanding and determining its *in-vivo* efficacy.

In conclusion, the antioxidant and anti-inflammatory potential of Myr, in addition to DPP-4 inhibition, could be the possible mechanism through which it exhibits anti-hyperglycemic effects. Though, the presence of HP lowers the modulatory effect of Myr, its ability to enhance antioxidant enzyme activities and reduce oxidative stress needs further experimental clarification. Further studies on the insulin signaling events in muscle and adipose tissue, under diabetic conditions, need to be conducted to assess the modulatory effects that Myr may possess other than the DPP-4 inhibition properties.

## Supporting information

S1 Checklist The ARRIVE guidelines checklist. (PDF)

S1 Fig Summary of the effect of Myr and HP, singly and in combination on the markers of hyperglycemia, and oxidative stress. Effect of high fat diet along with low doze of STZ on the markers are shown on the left. Effect of Myr, HP and their combination are shown on the right.
(TIF)

S1 Raw data (XLSX)

S1 Data Protein expression blots. (PDF)
