# Supplementary figures and images for: Anti-hyperglycemic activity of myricetin, through inhibition of DPP-4 and enhanced GLP-1 levels, is attenuated by co-ingestion with lectin-rich protein
Source: PLoS One. 2020 Apr 13;15(4):e0231543. doi: 10.1371/journal.pone.0231543 (PMC7153899; doi:10.1371/journal.pone.0231543)

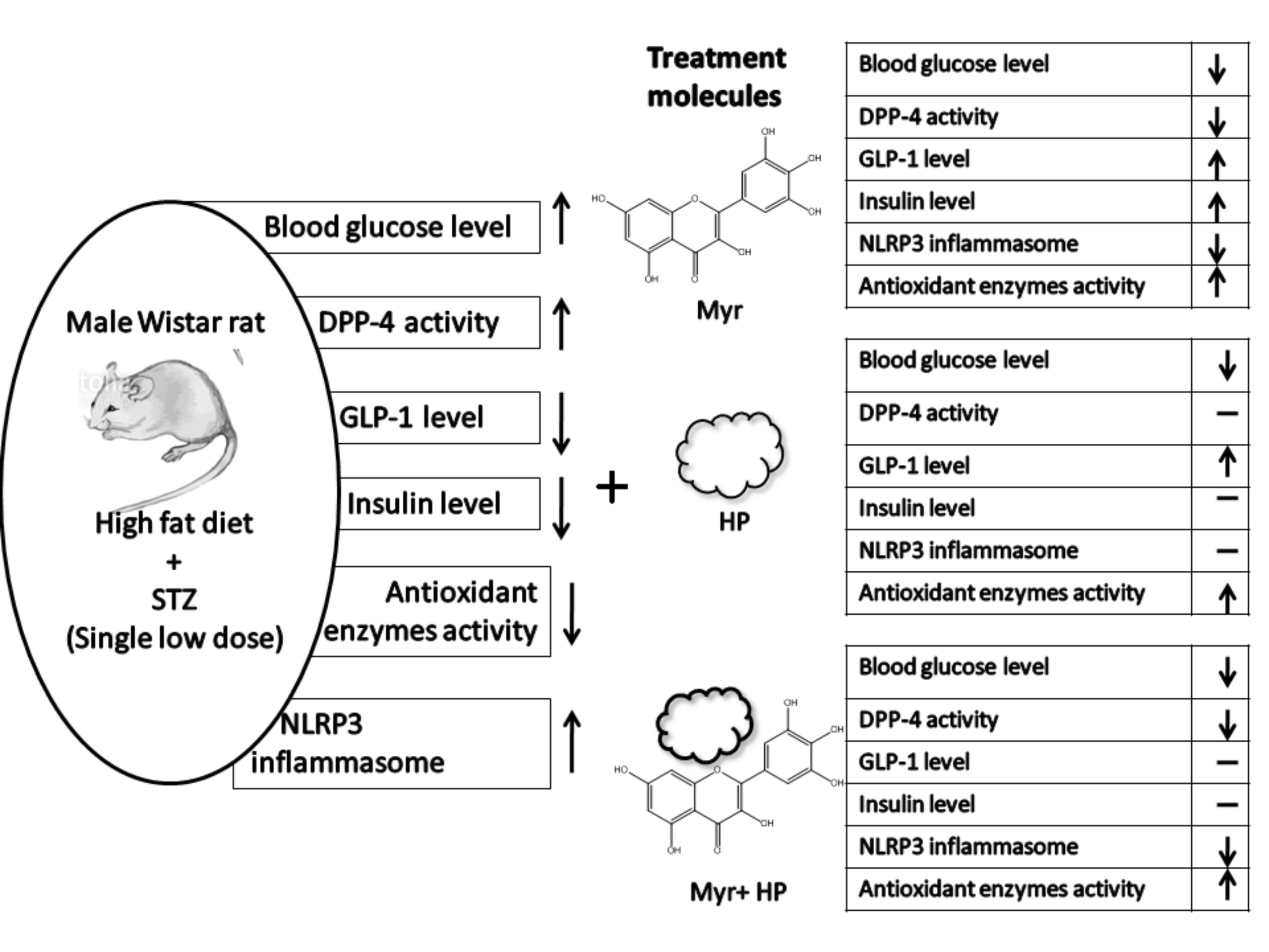

Supplement: S1 Fig — Effect of high fat diet along with low doze of STZ on the markers are shown on the left. Effect of Myr, HP and their combination are shown on the right. (TIF) [file pone.0231543.s002.tif]
