# Supplementary material for: Anti-hyperglycemic activity of myricetin, through inhibition of DPP-4 and enhanced GLP-1 levels, is attenuated by co-ingestion with lectin-rich protein
Source: PLoS One. 2020 Apr 13;15(4):e0231543. doi: 10.1371/journal.pone.0231543 (PMC7153899; doi:10.1371/journal.pone.0231543)

GLP-1 protein expression Blots

Sample 1: GLP-1 Blot

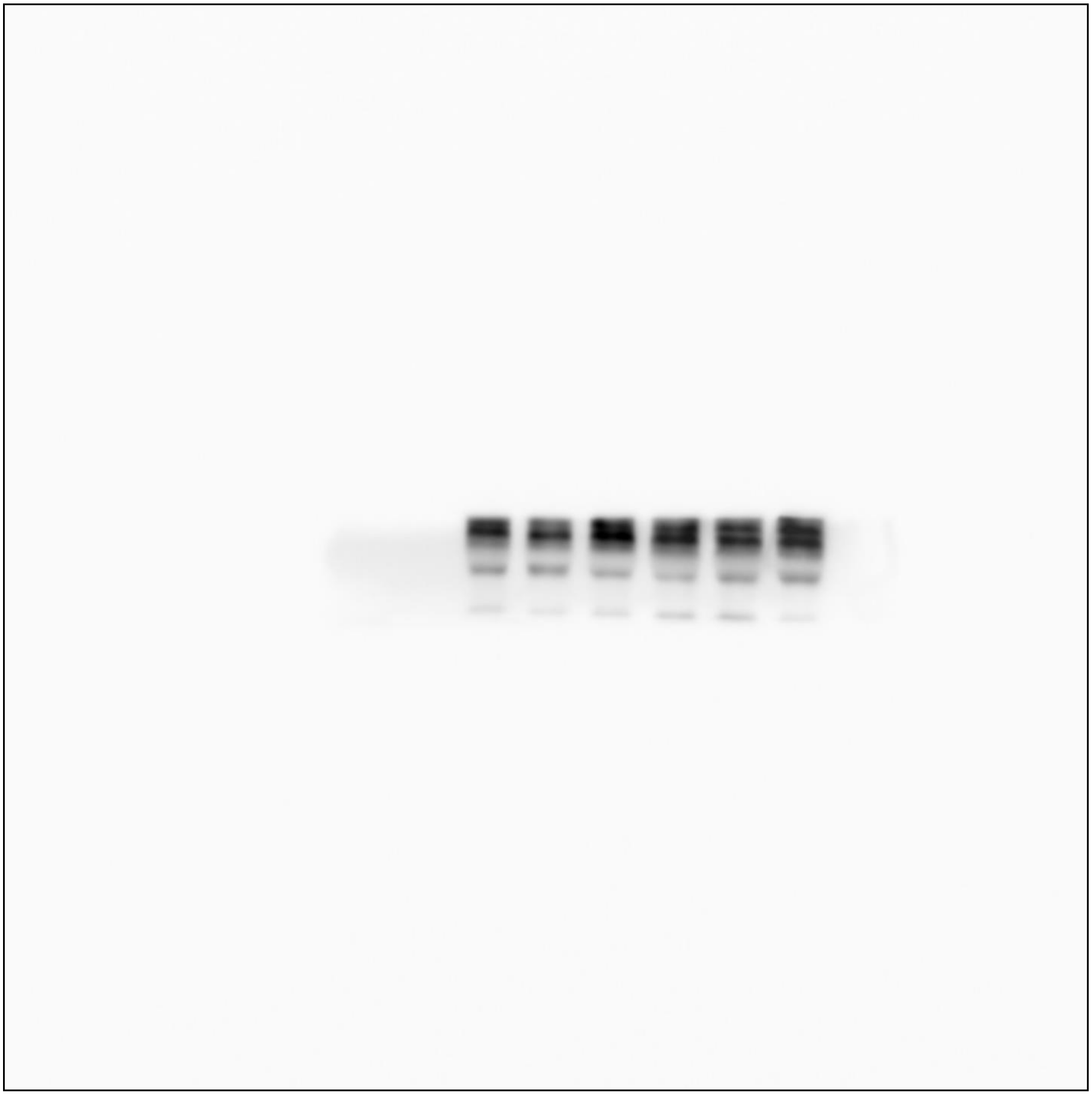

Sample 1: Plots of GLP1

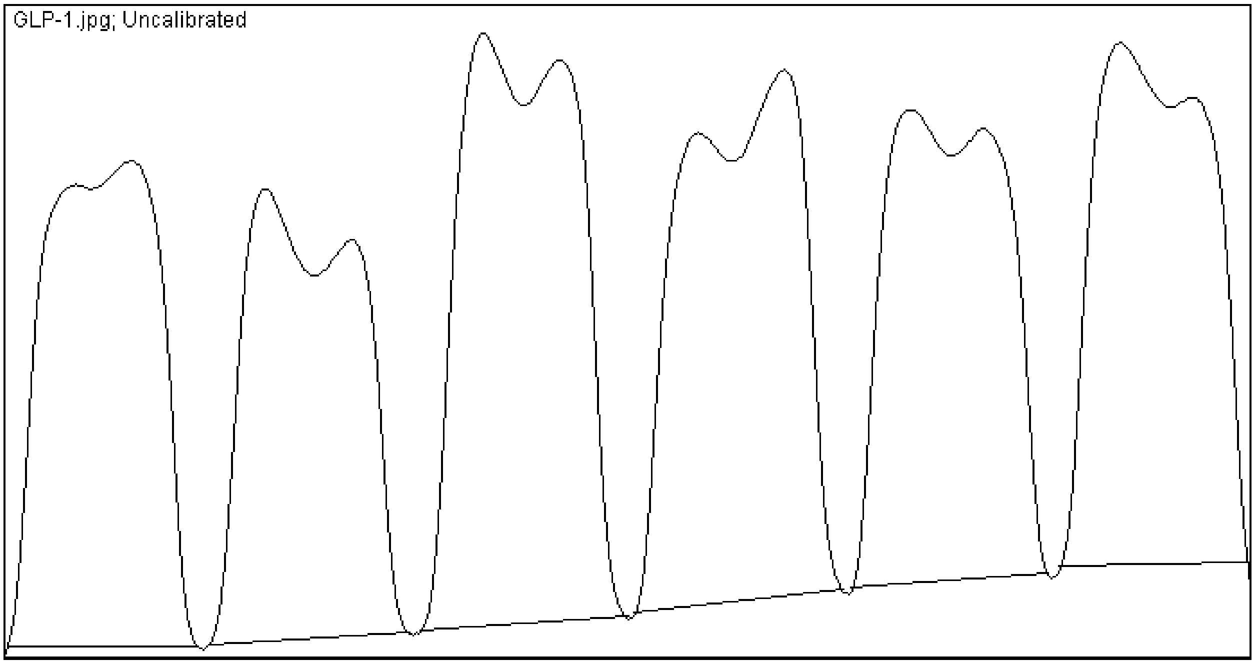

Sample 1: Transferrin Blot

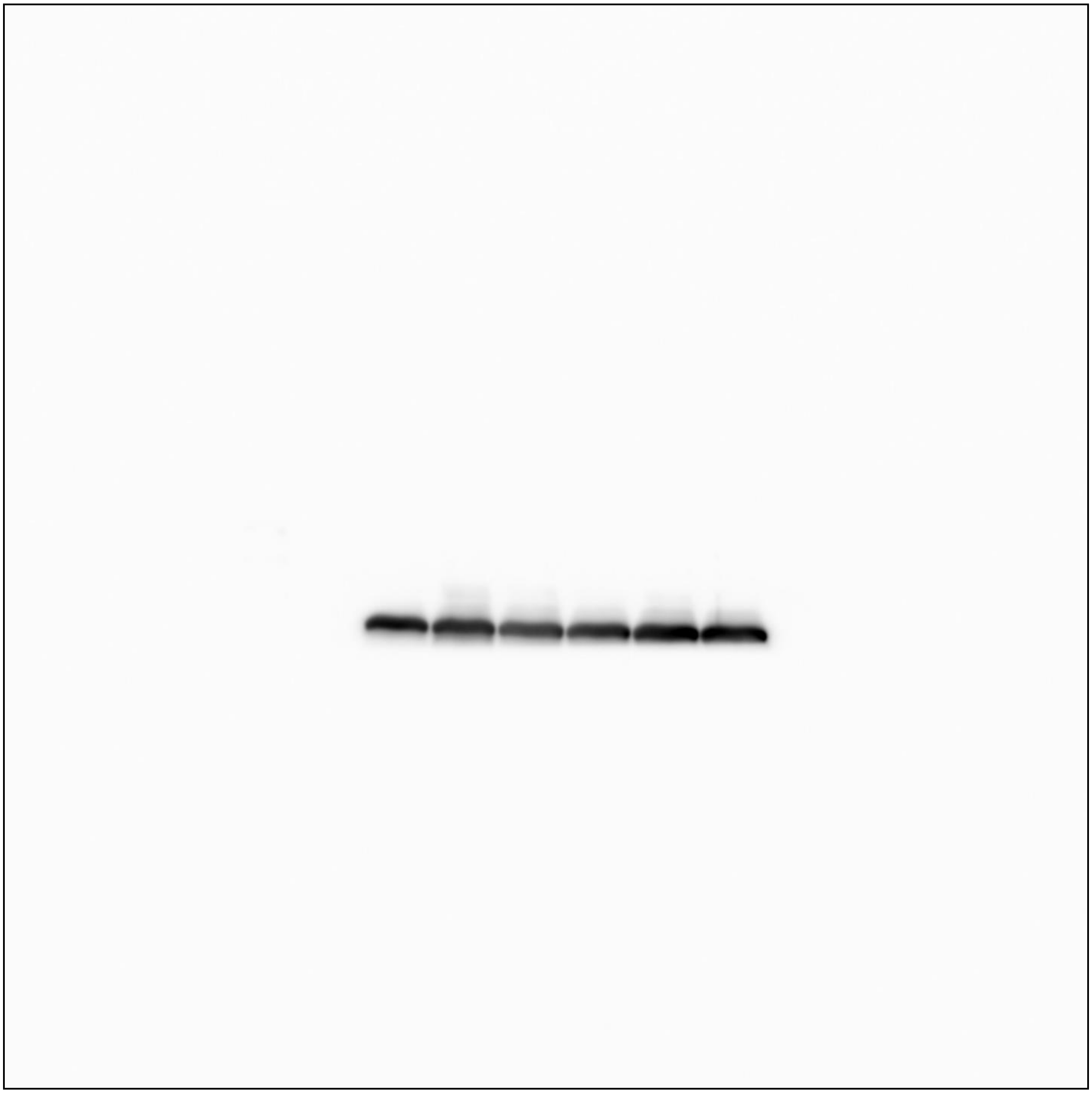

Sample 1: Plots of transferrin

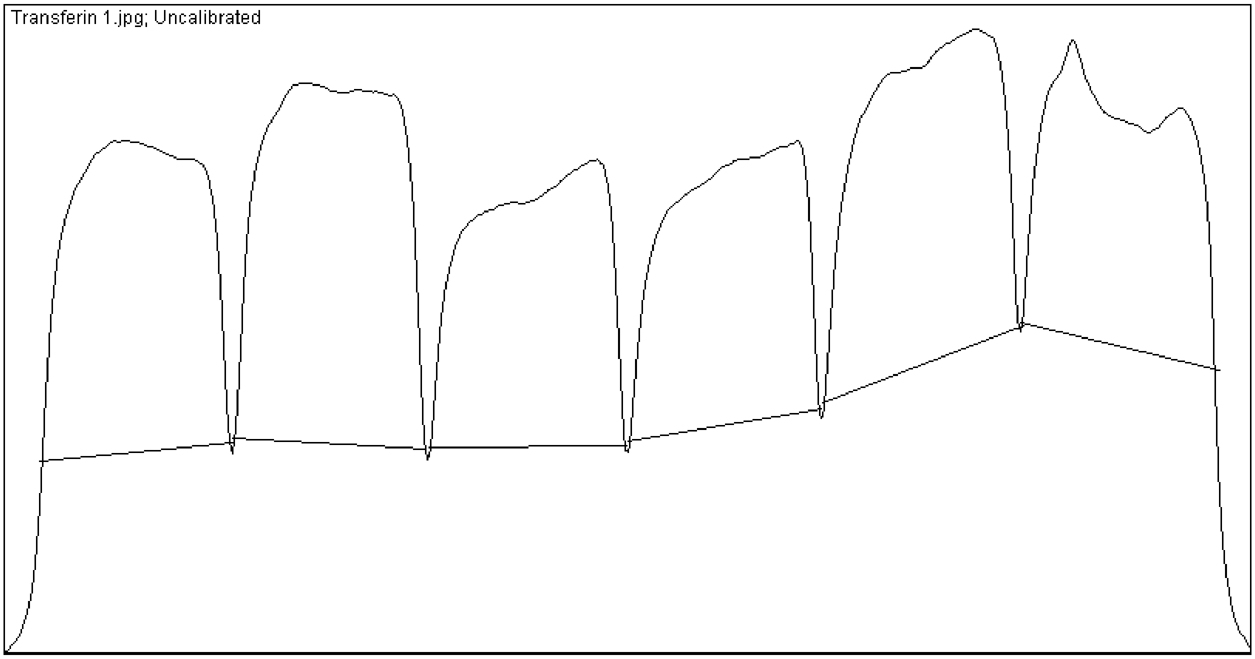

Sample 2: GLP-1 Blot

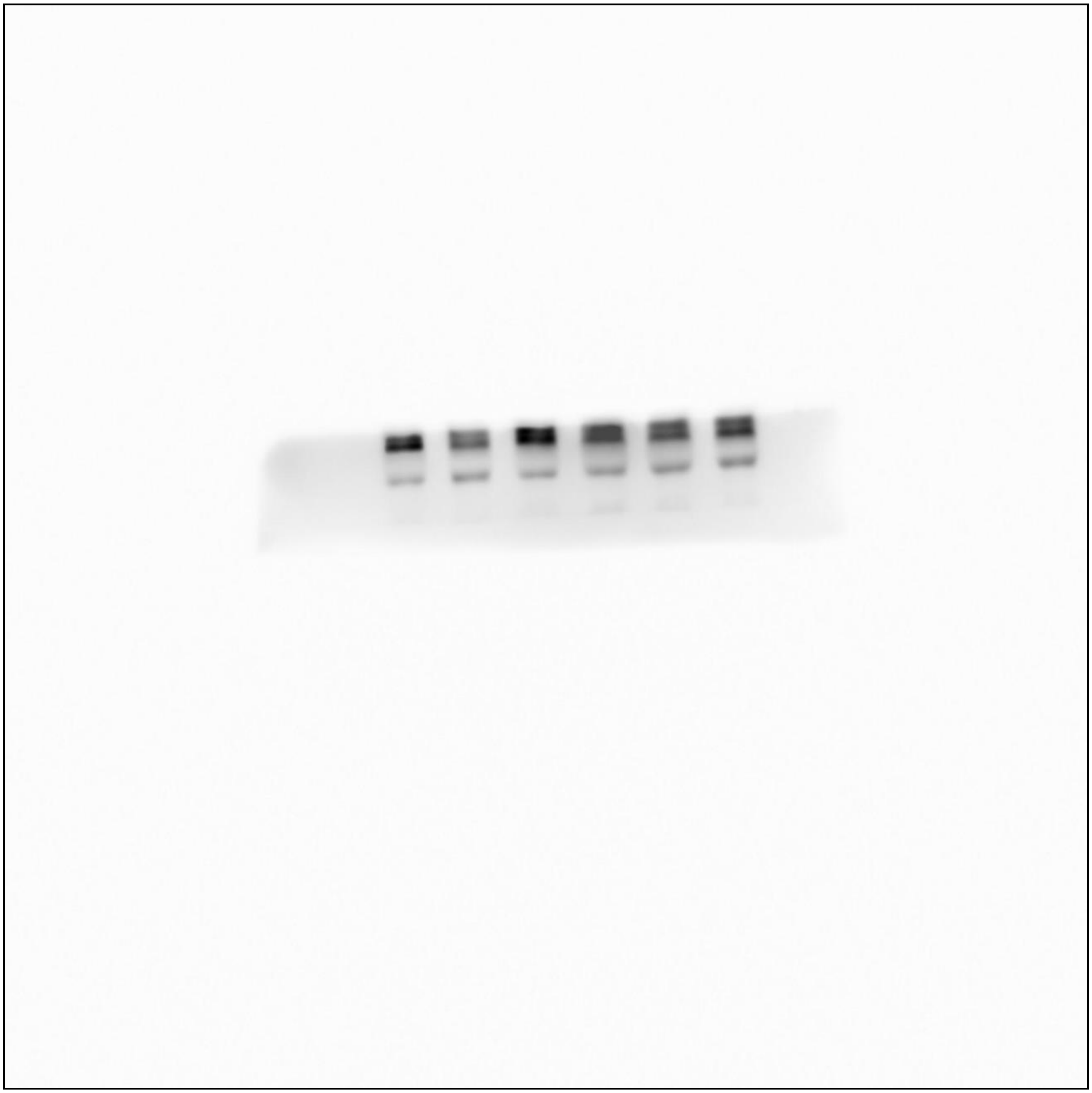

Sample 2: Plots of GLP-1

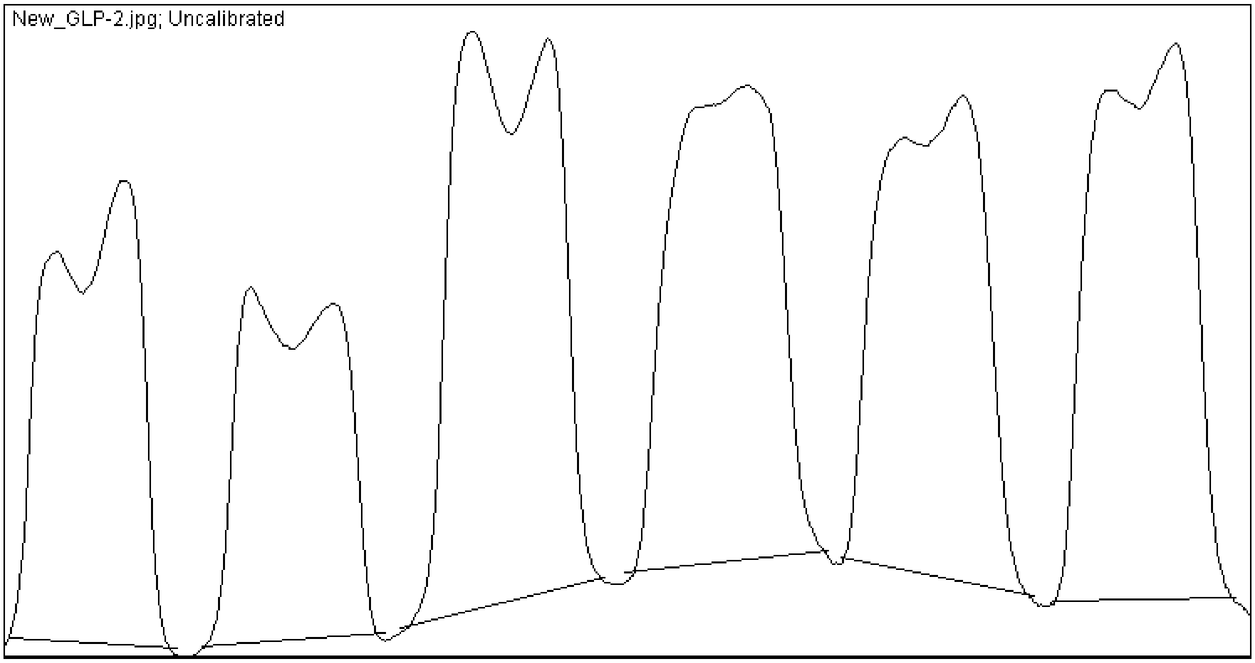

Sample 2: Transferrin Blot

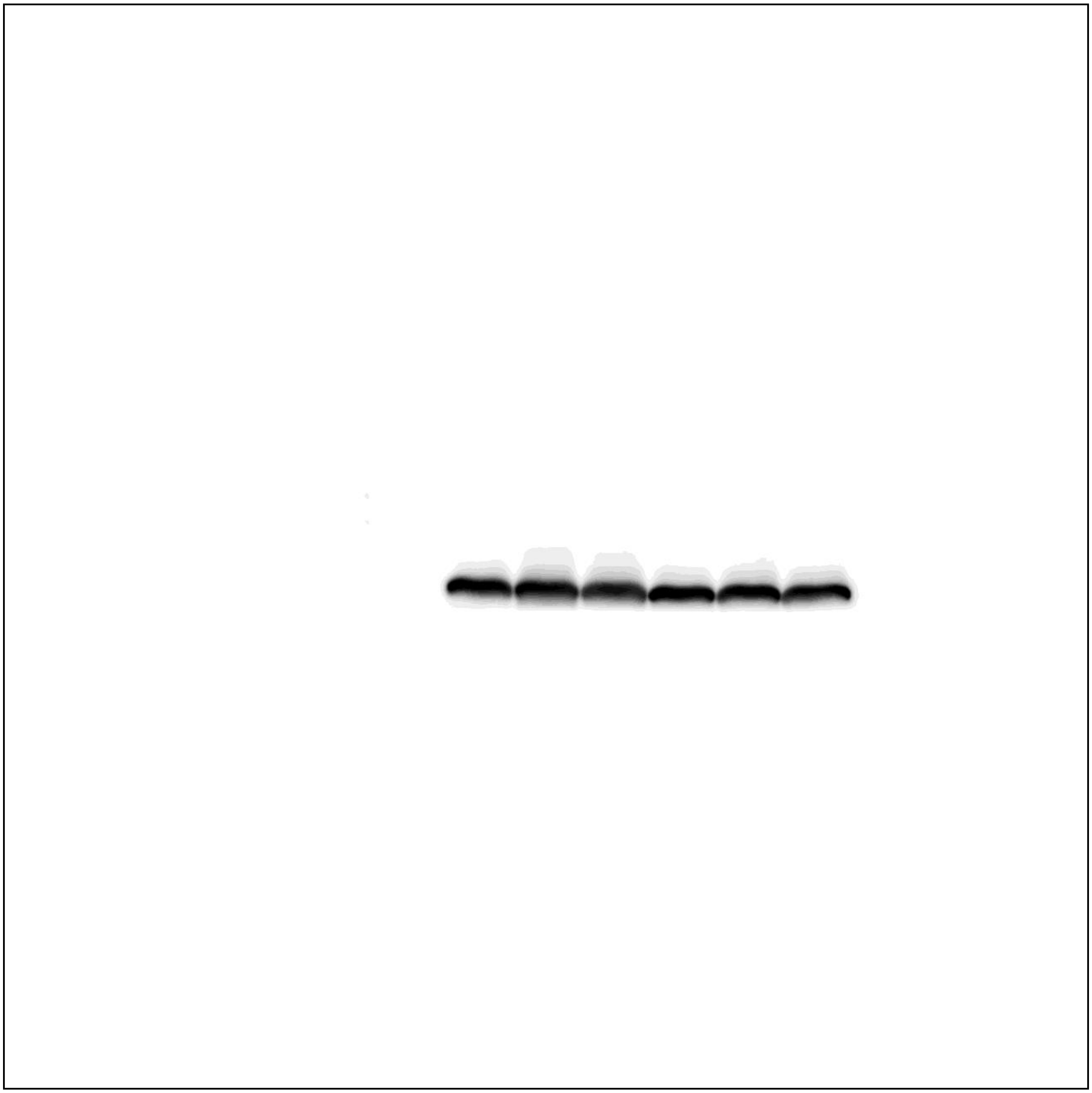

Sample 2: Plots of Transferrin

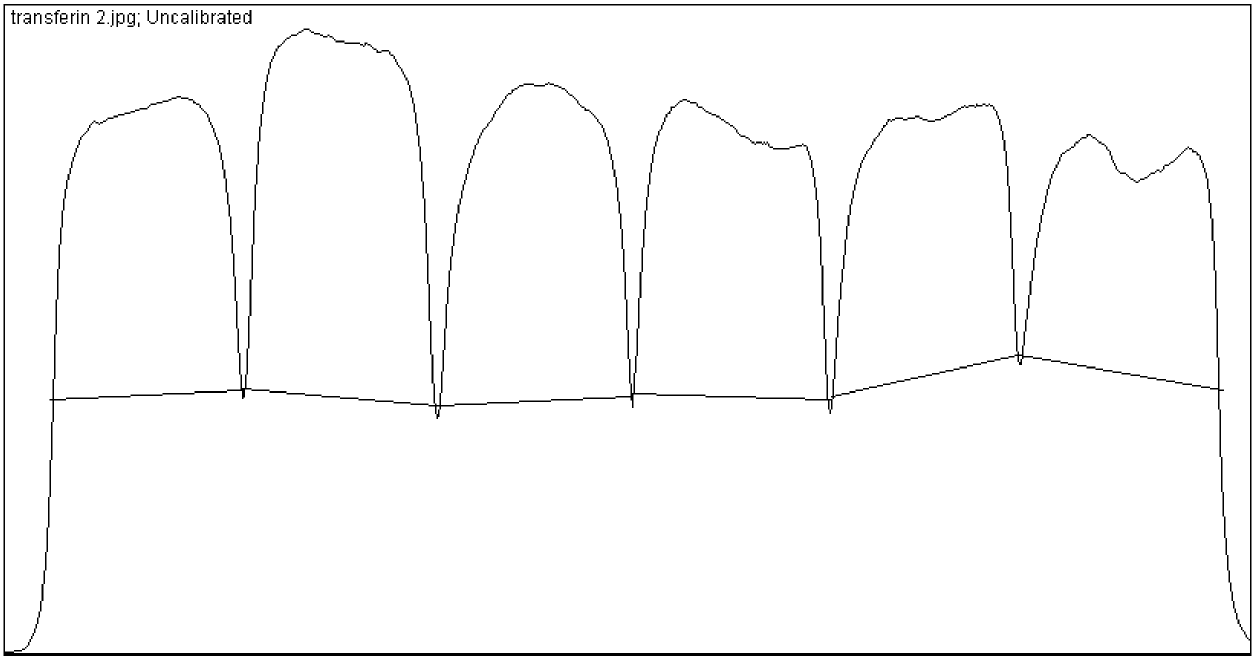

Sample 3- GLP-1 Blot

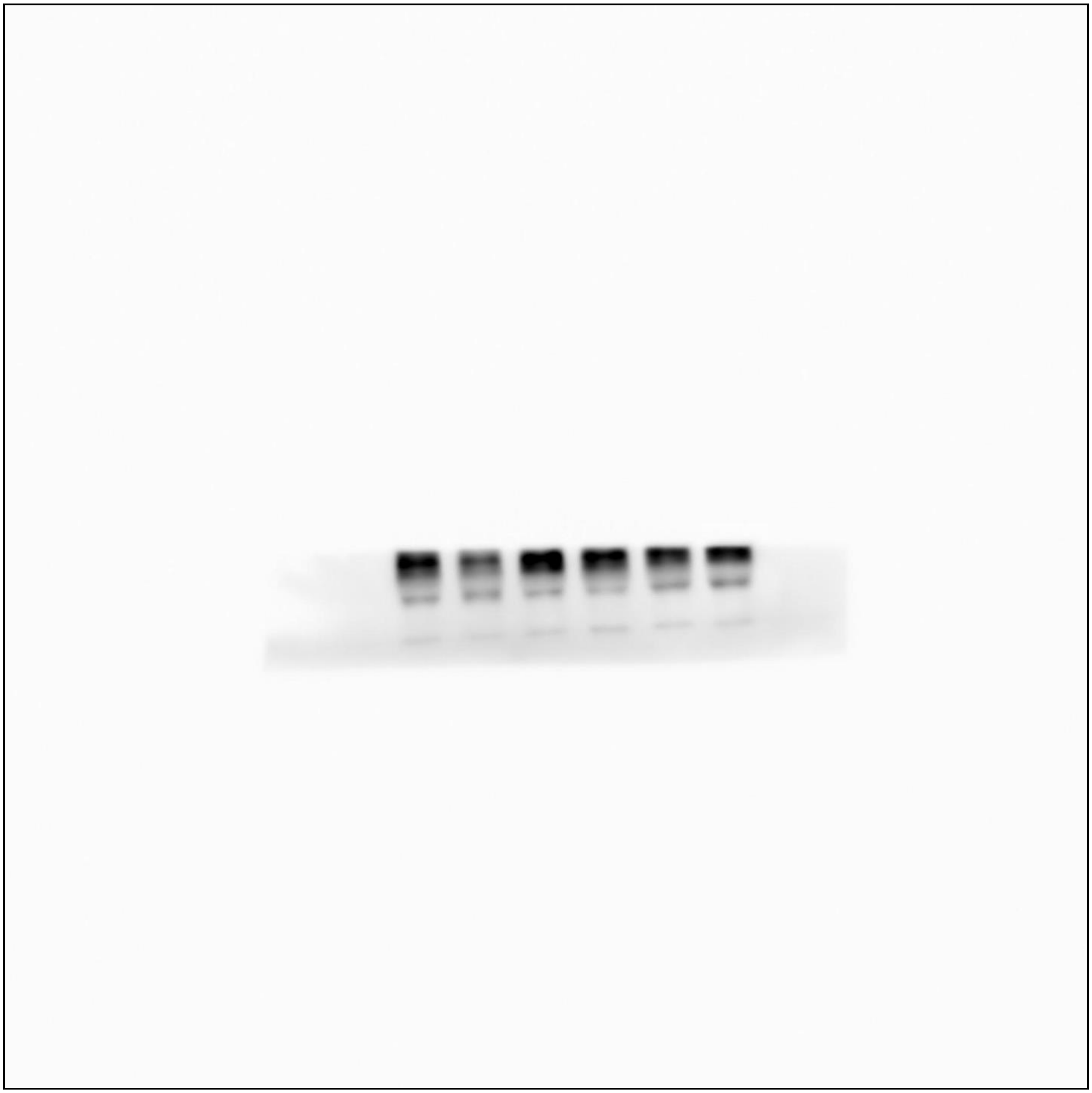

Sample 3: Plots of GLP-1

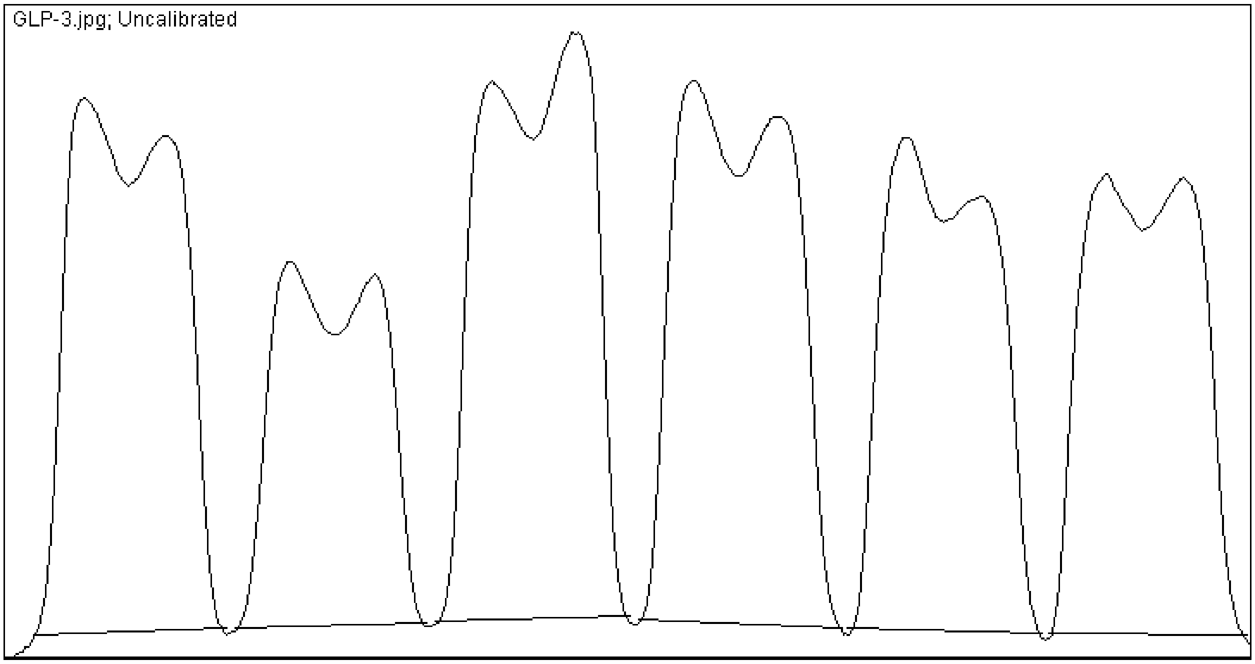

Sample 3: Transferrin Blot

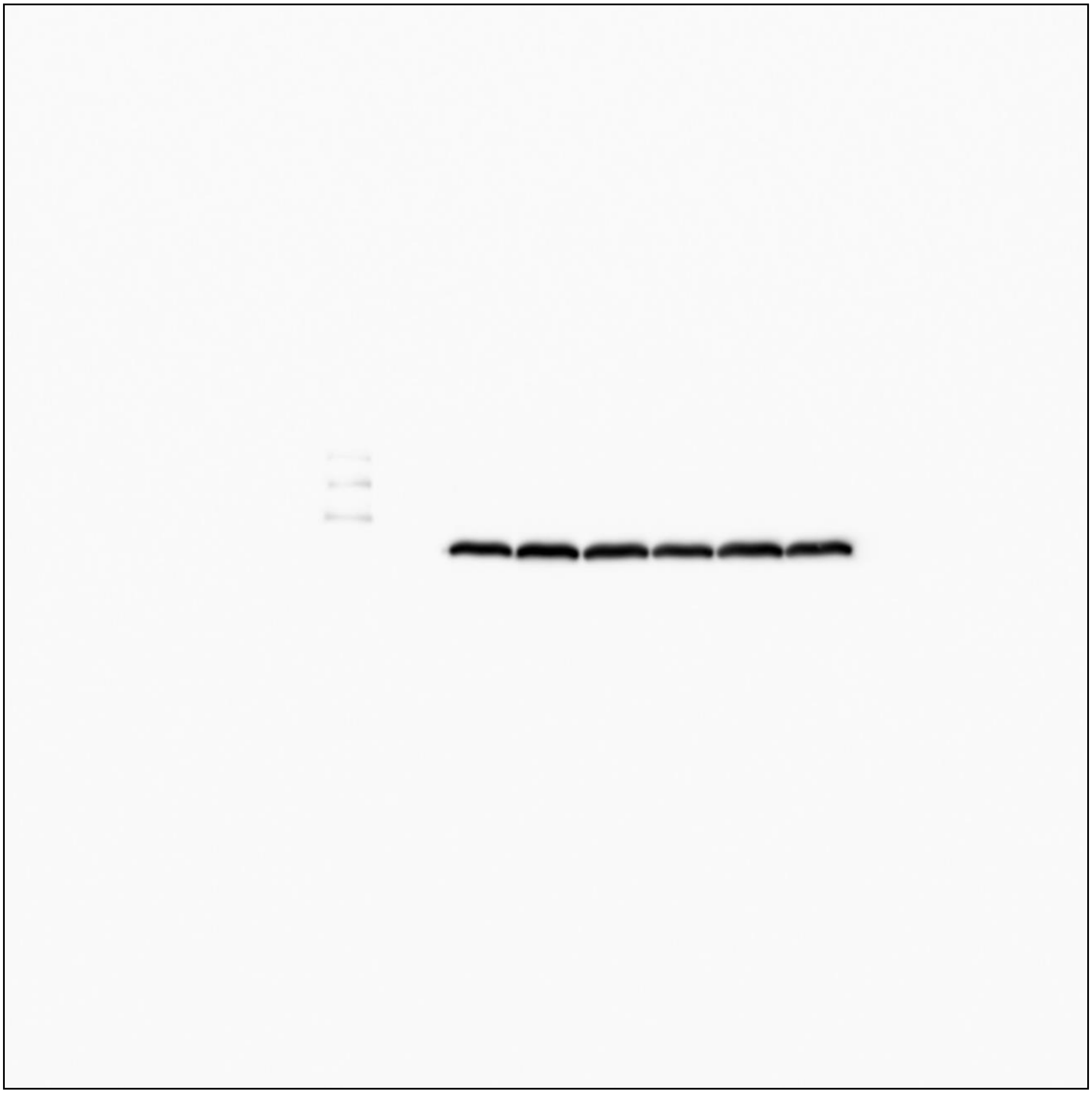

Sample 3: Plots of Transferrin

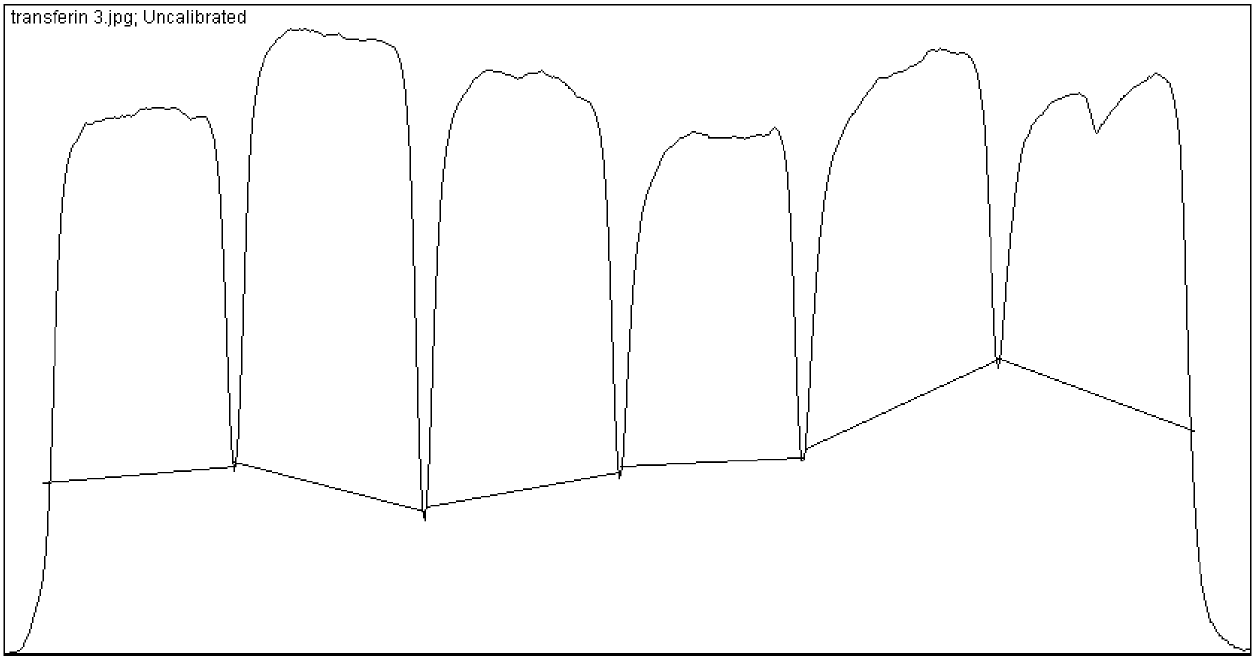

Sample 4: GLP-1 Blot

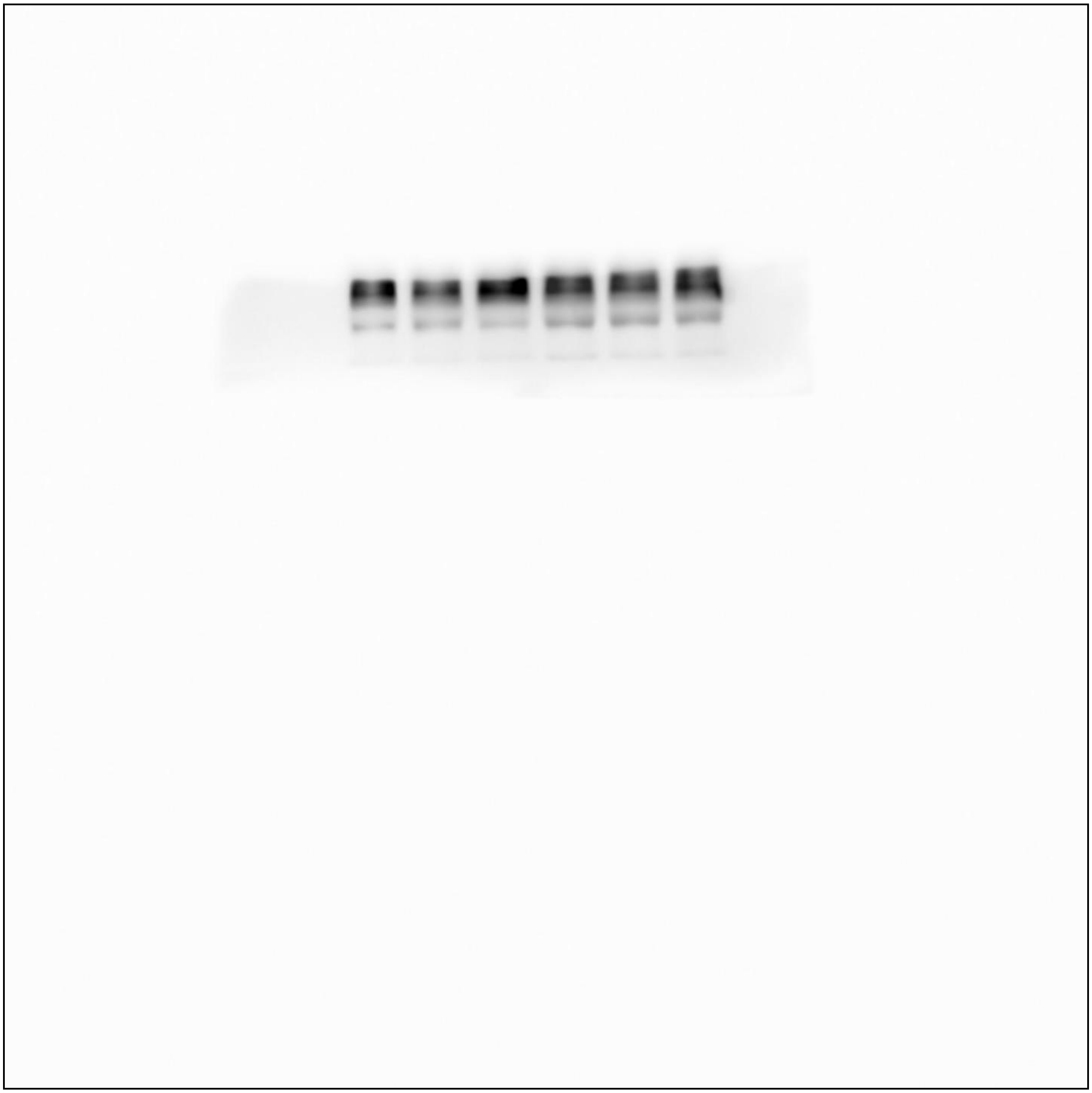

Sample 4: Plots of GLP-1

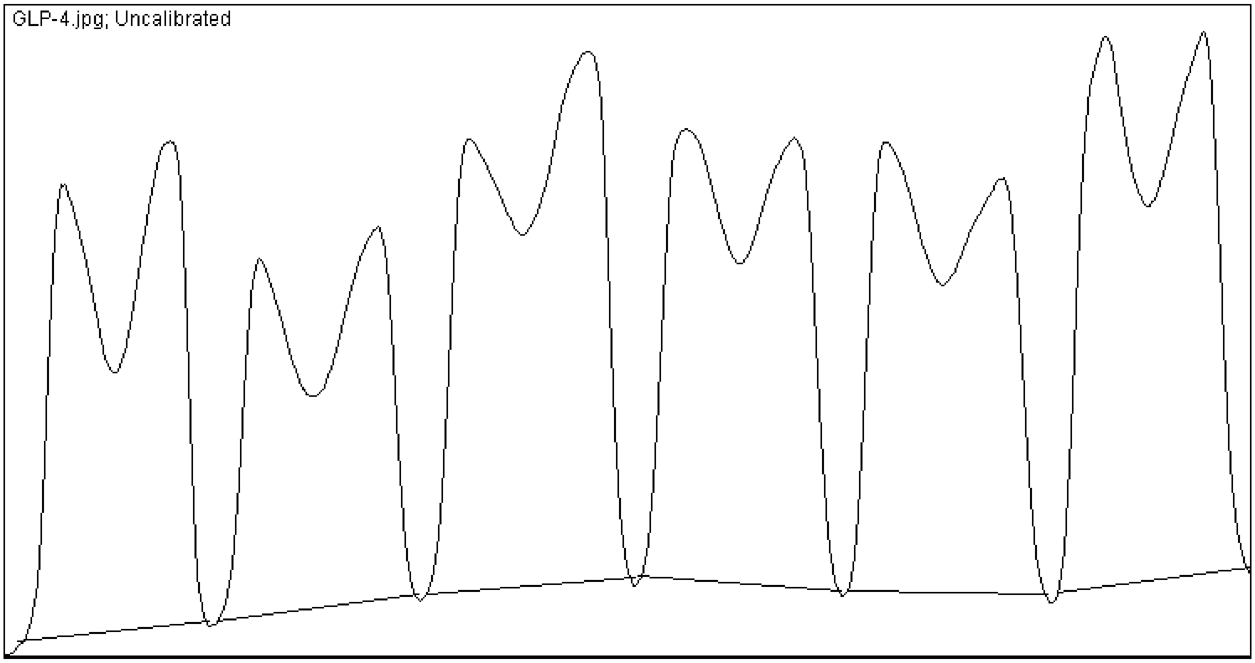

Sample 4: Transferrin Blot

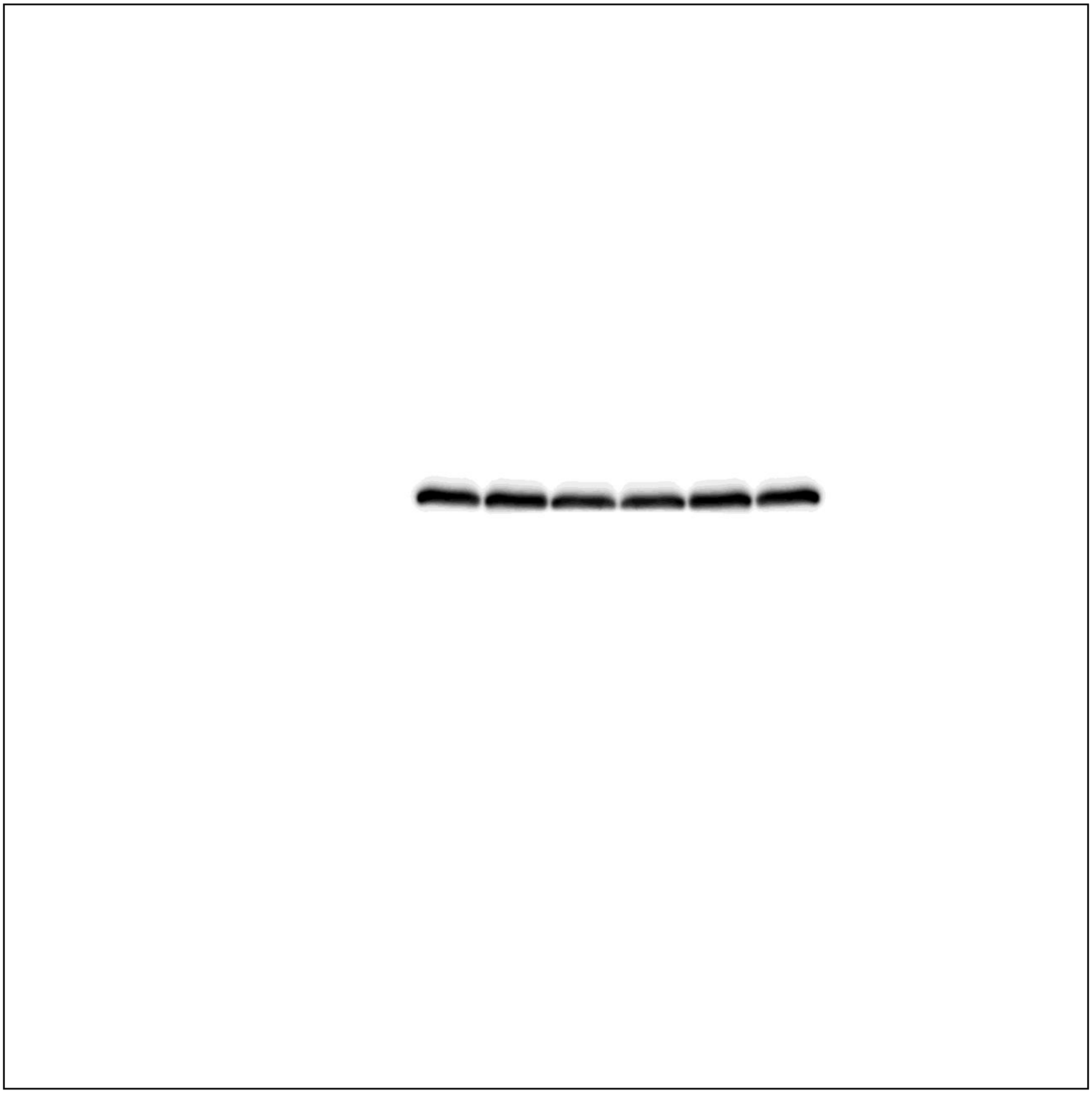

Sample 4: Plots of Transferrin

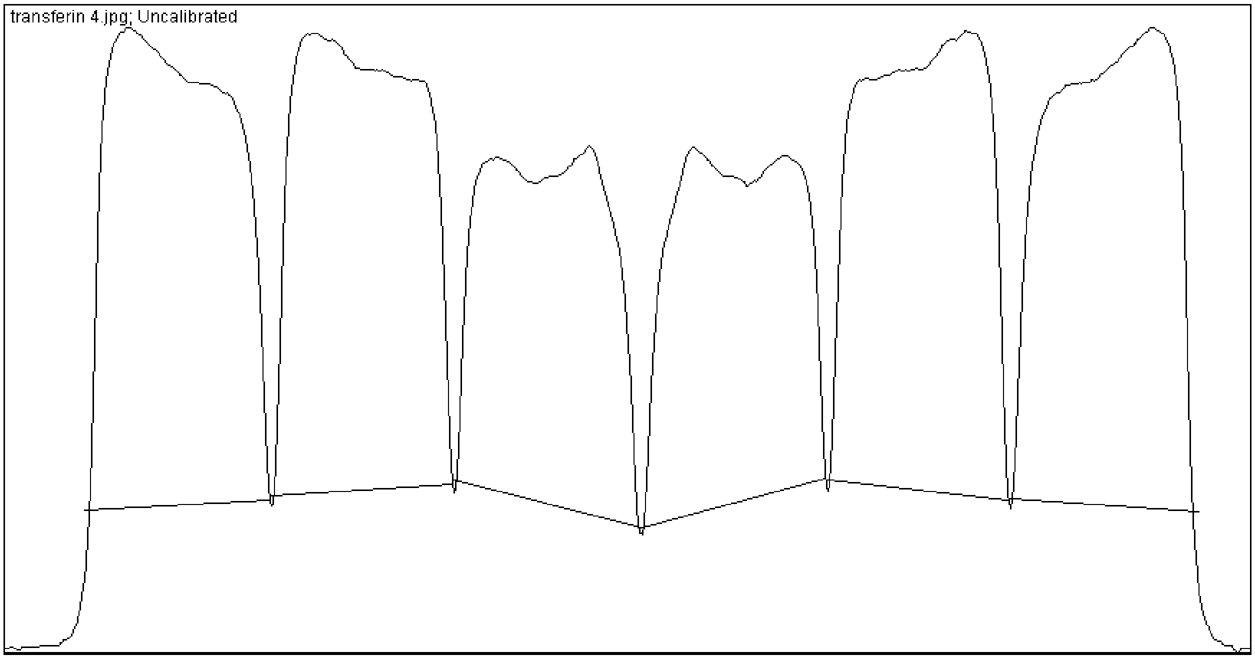

Sample 5: GLP-1 Blot

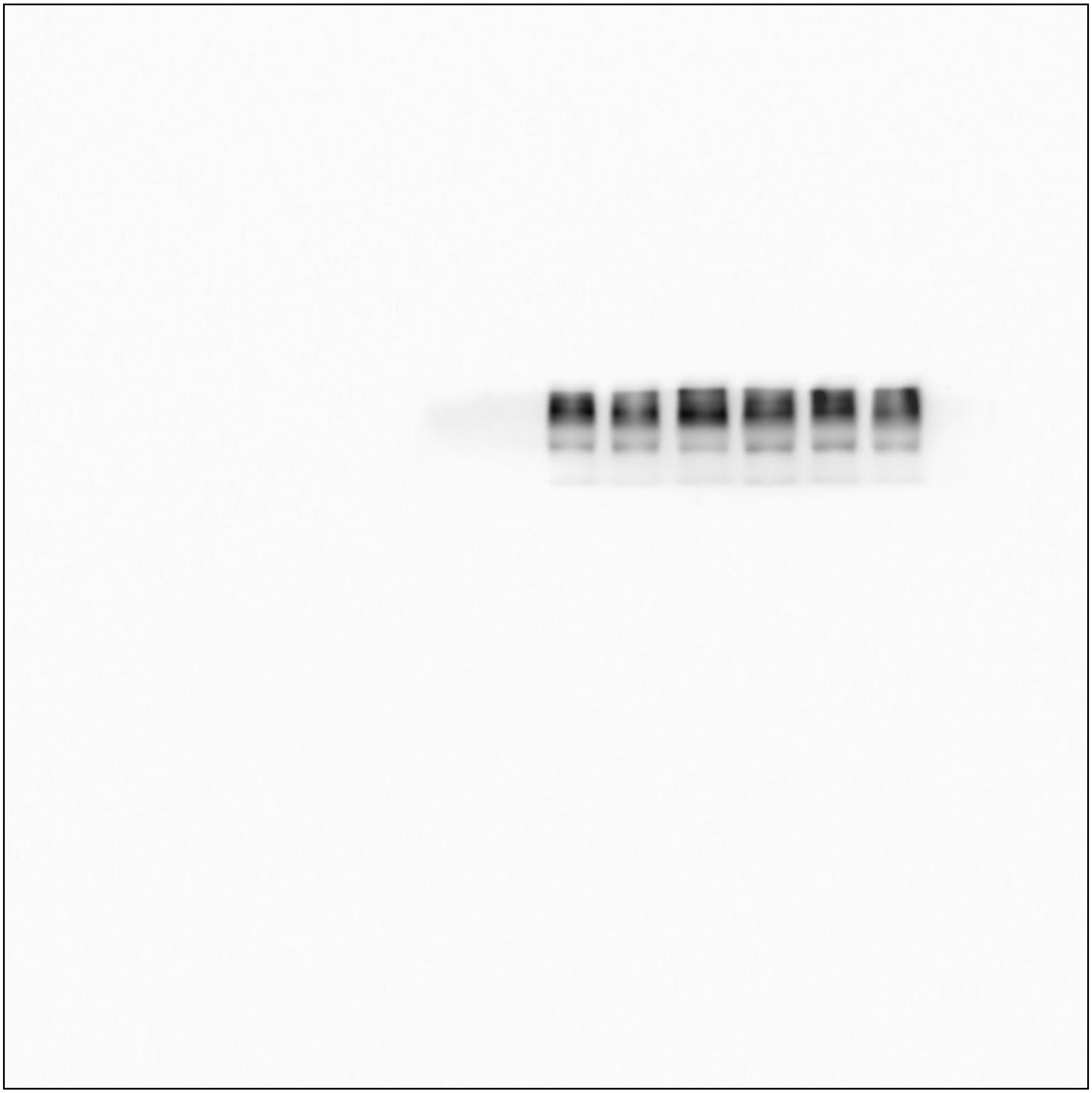

Sample 5: Plots of GLP-1

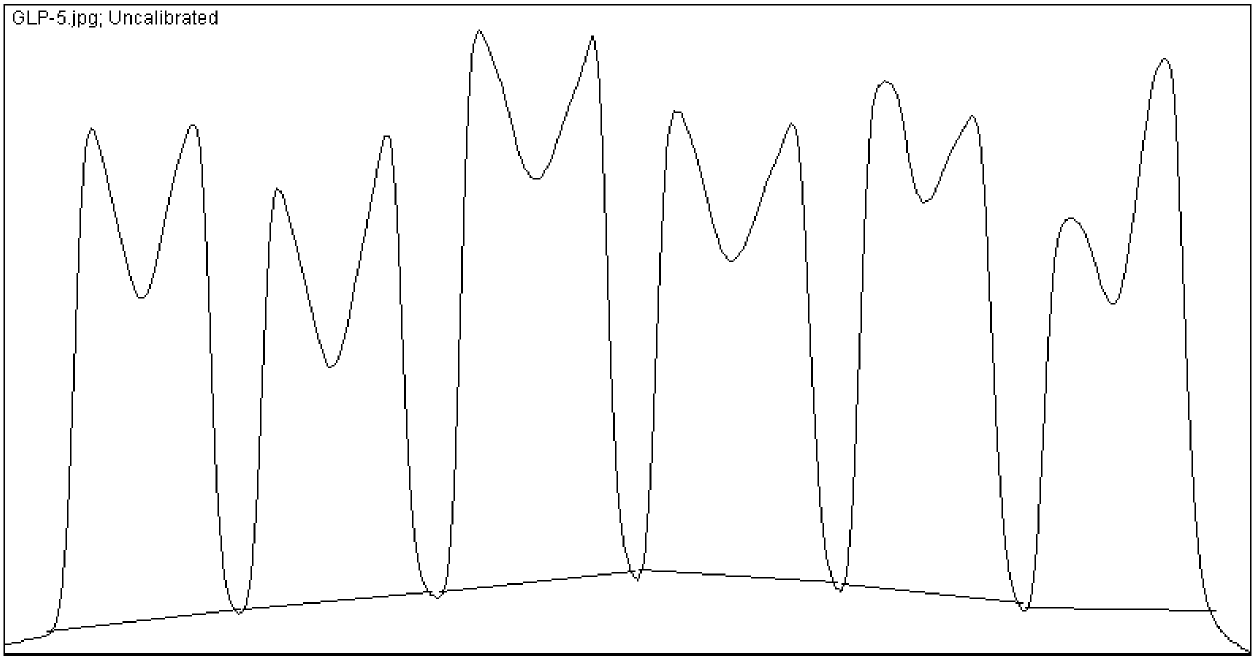

Sample 5: Transferrin Blot

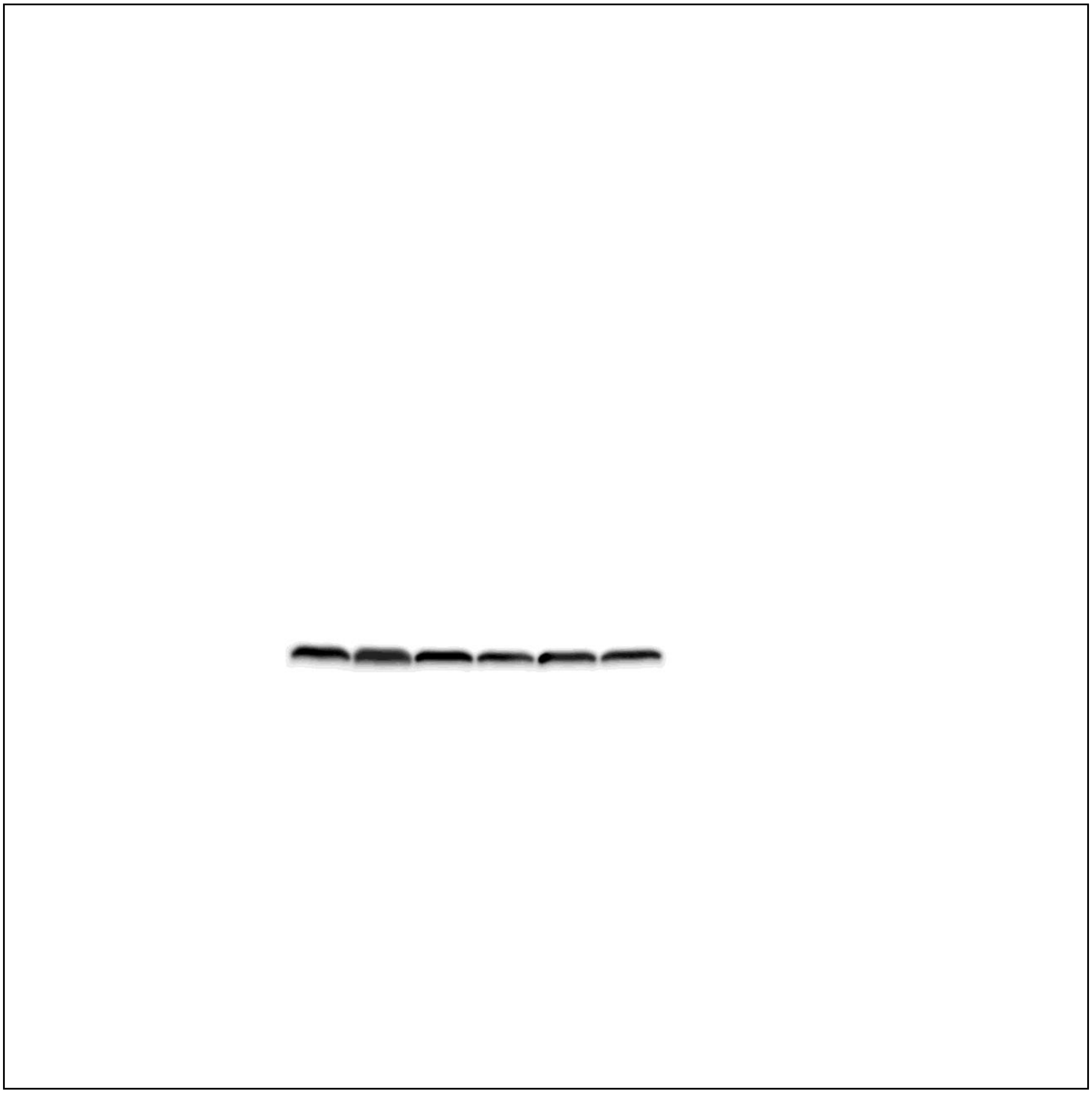

Sample 5: Plots of Transferrin

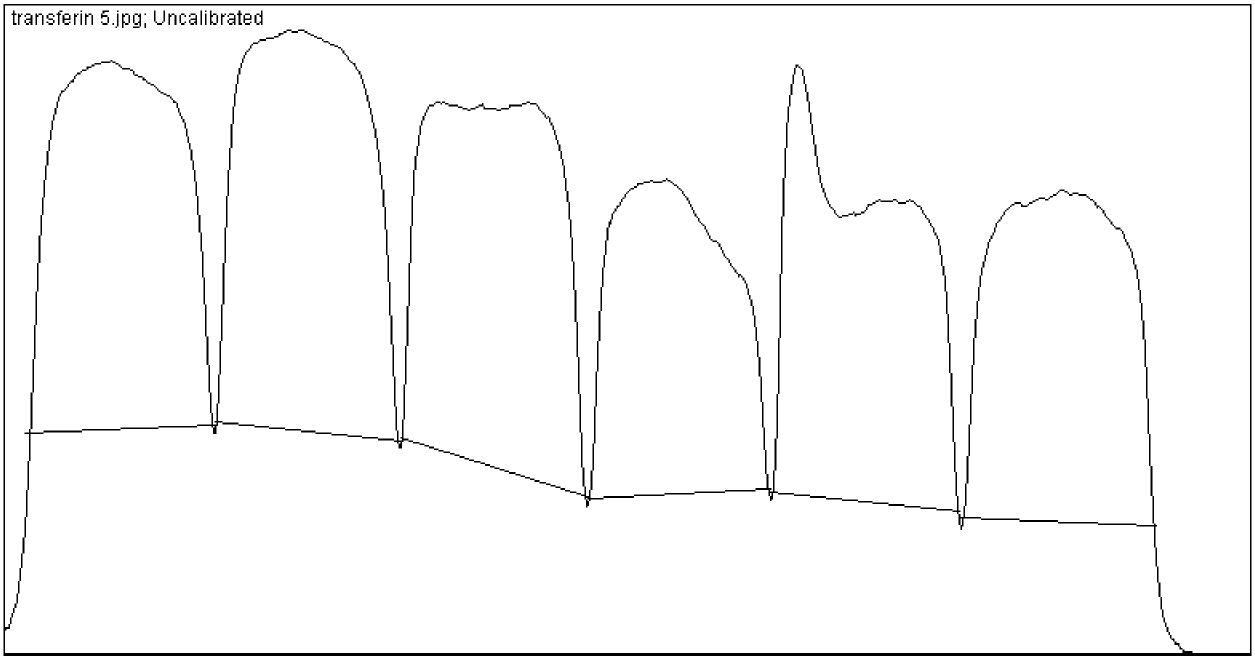

Sample 6: GLP-1 Blot

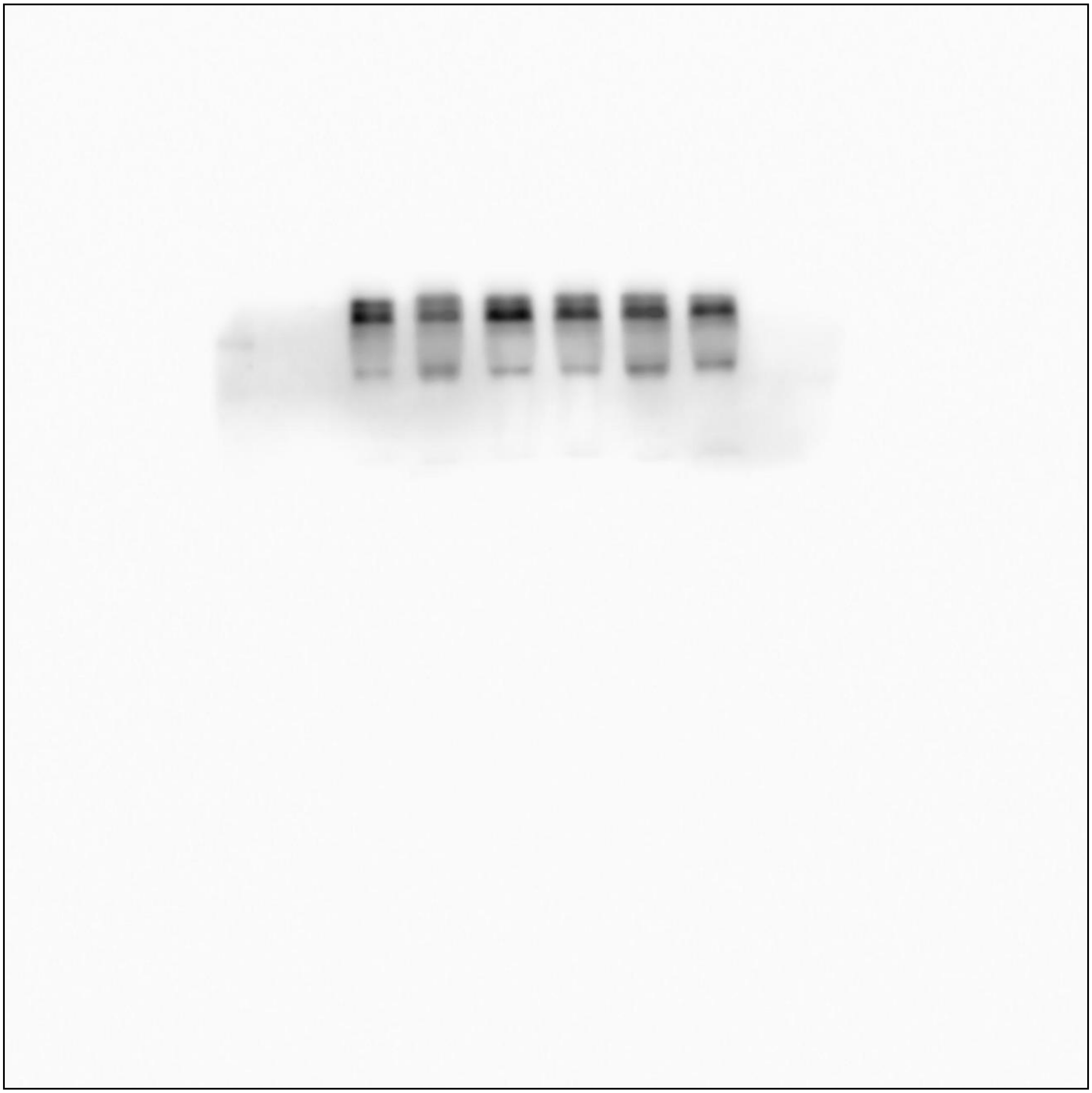

Sample 6: Plots of GLP-1

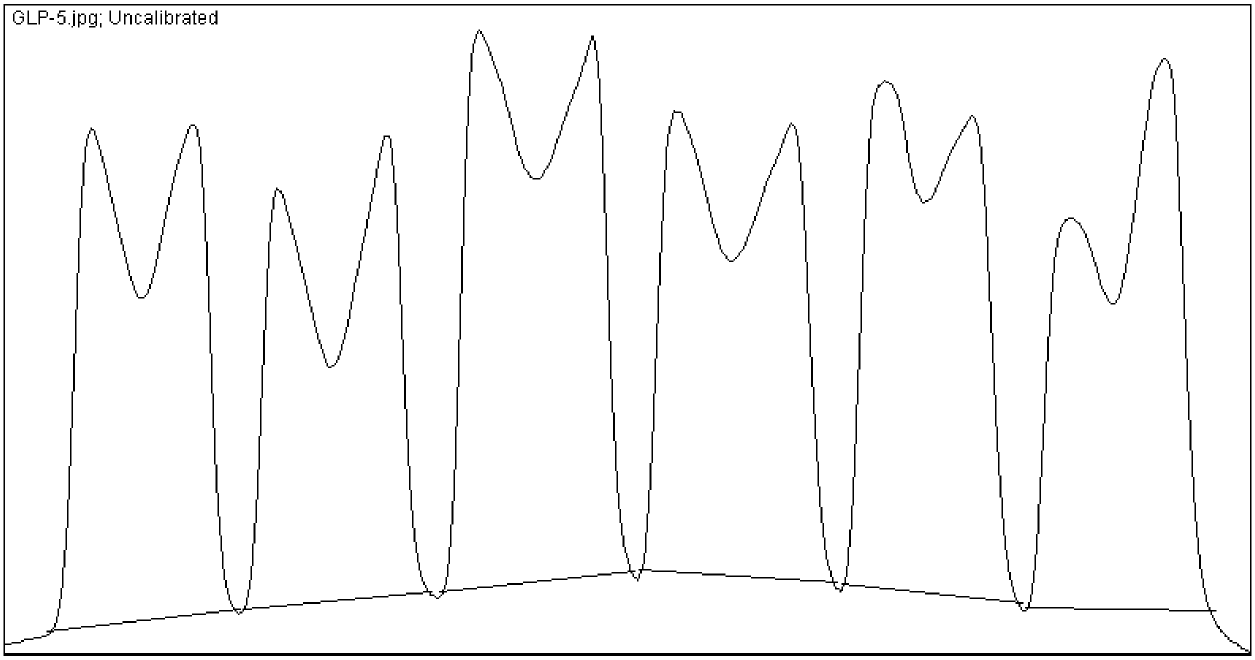

Sample 6: Transferrin Blot

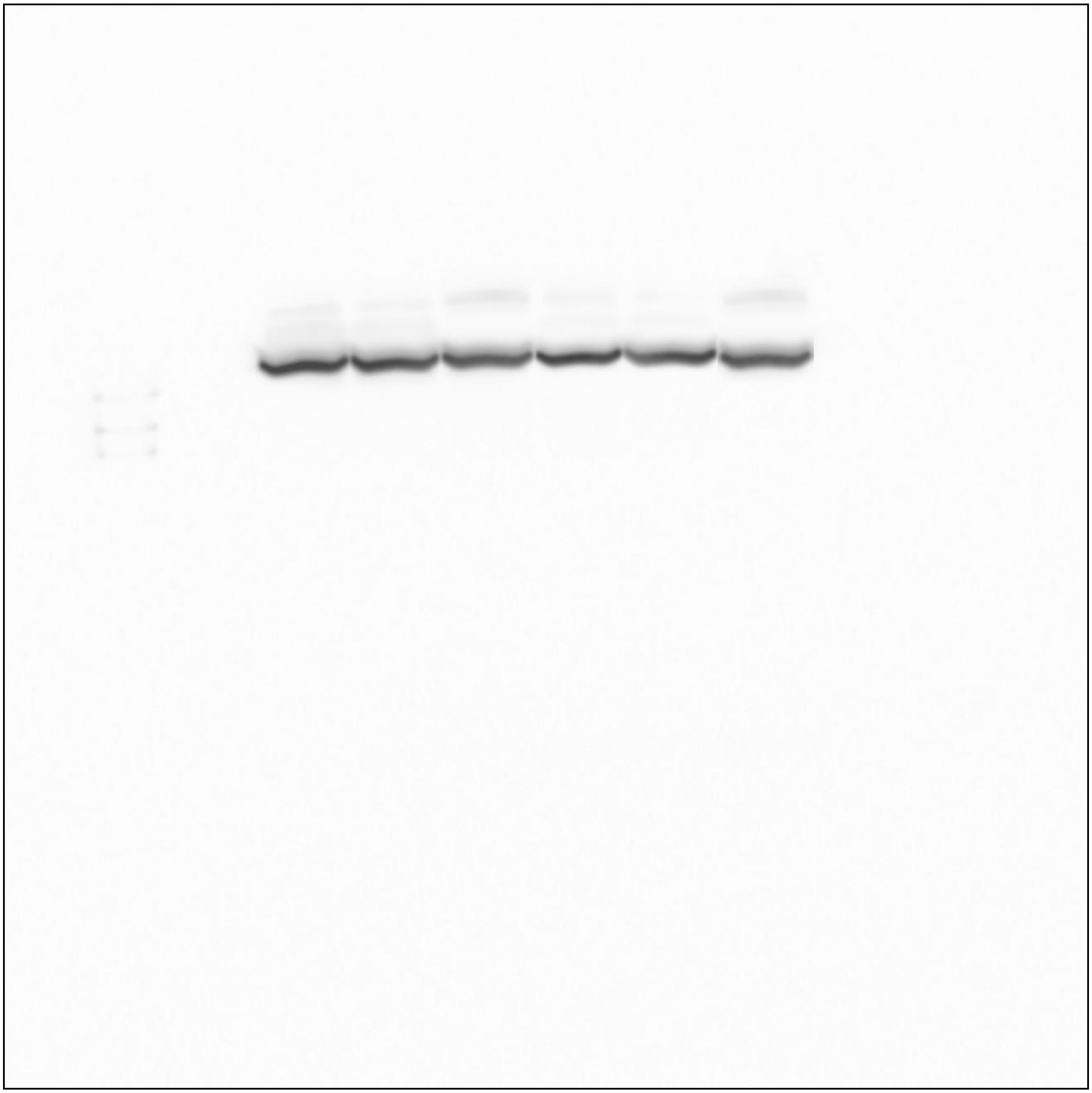

Sample 6: Plots of Transferrin

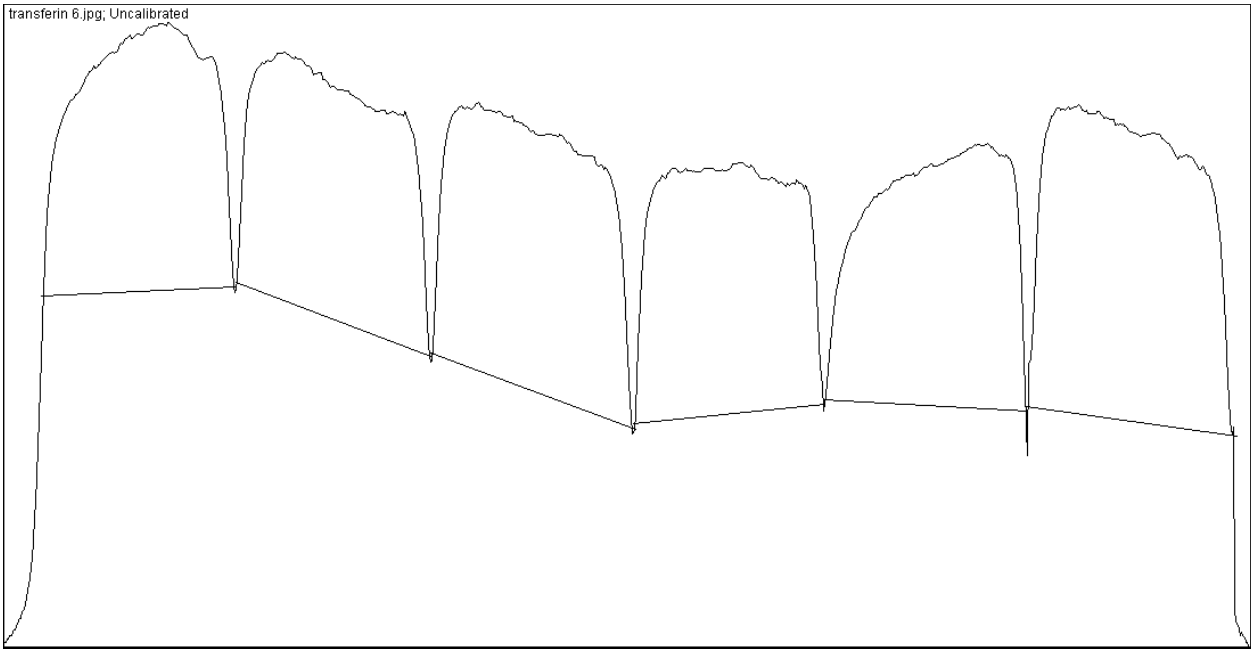

NLRP-3 Protein Expression Blots

Sample 1 & 2: NLRP3 Blot

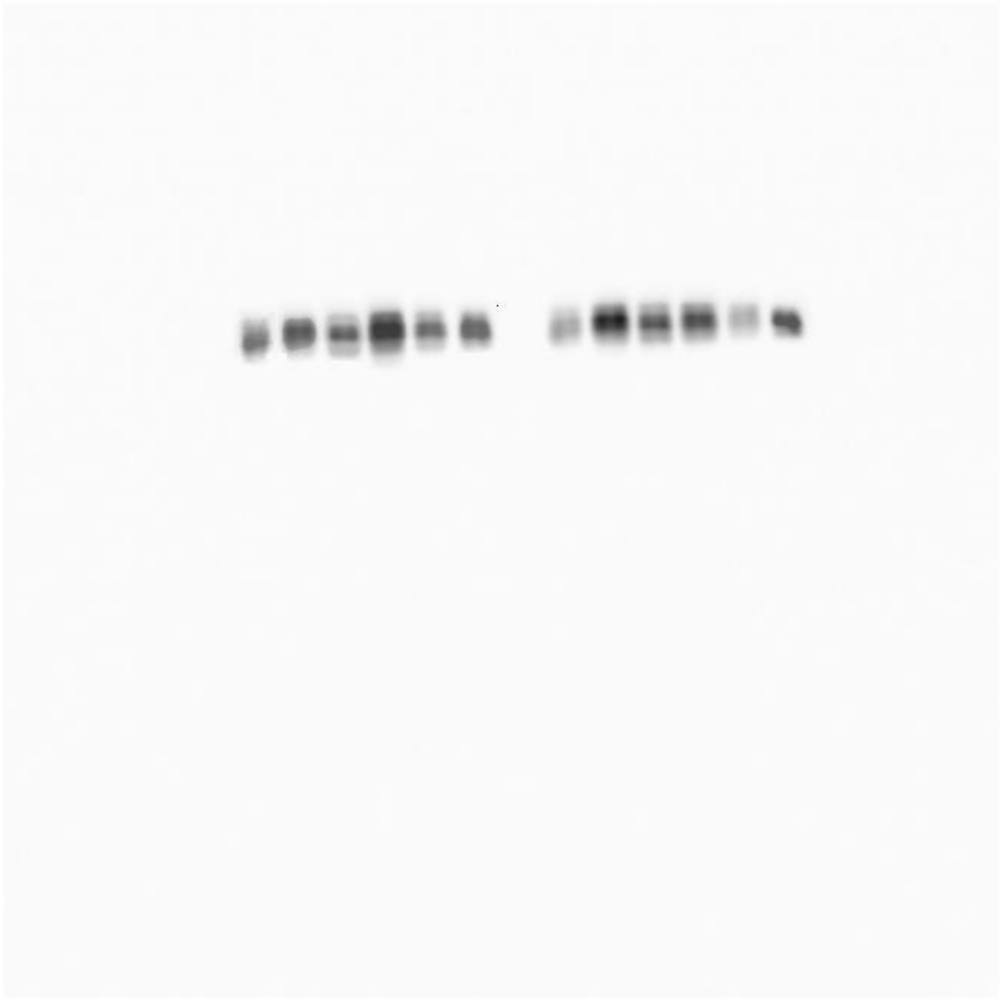

Sample 1 & 2: Plots of NLRP3

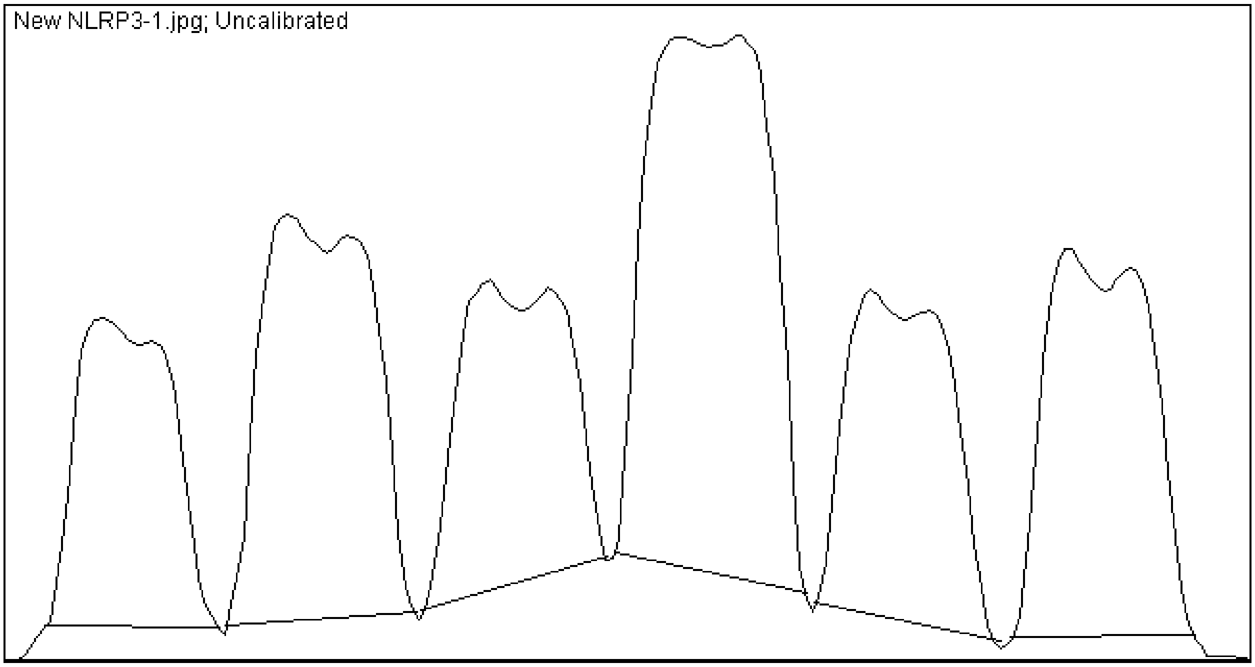

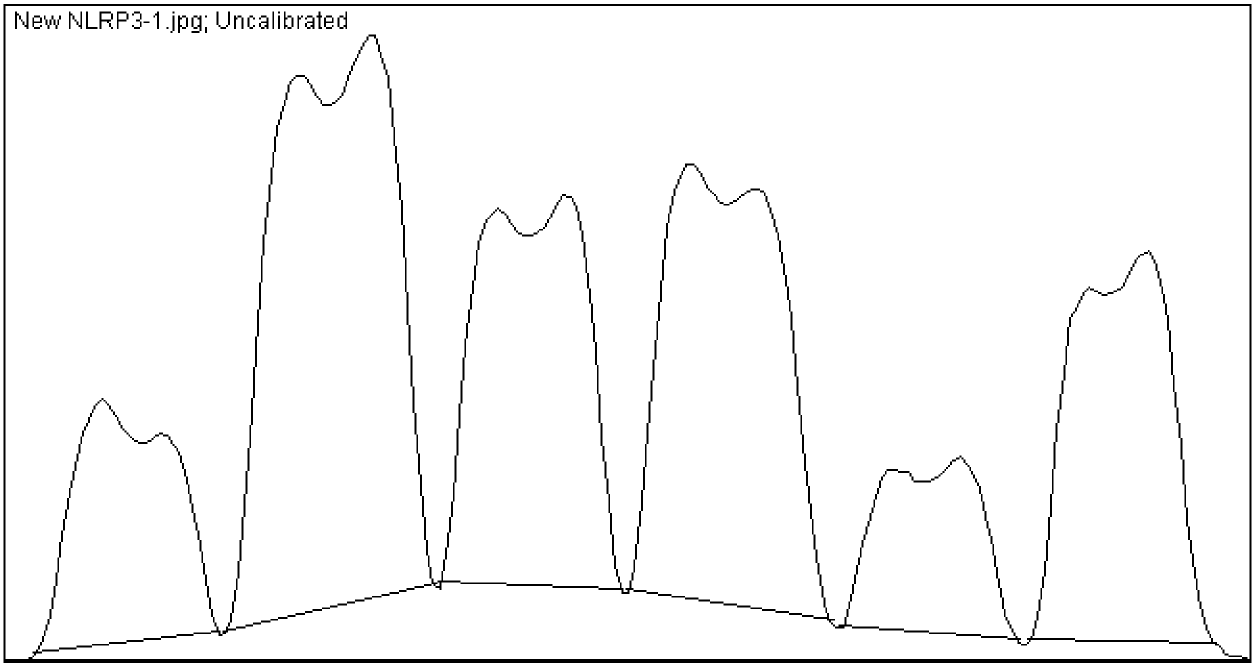

Sample 1 & 2: Tubulin Blot

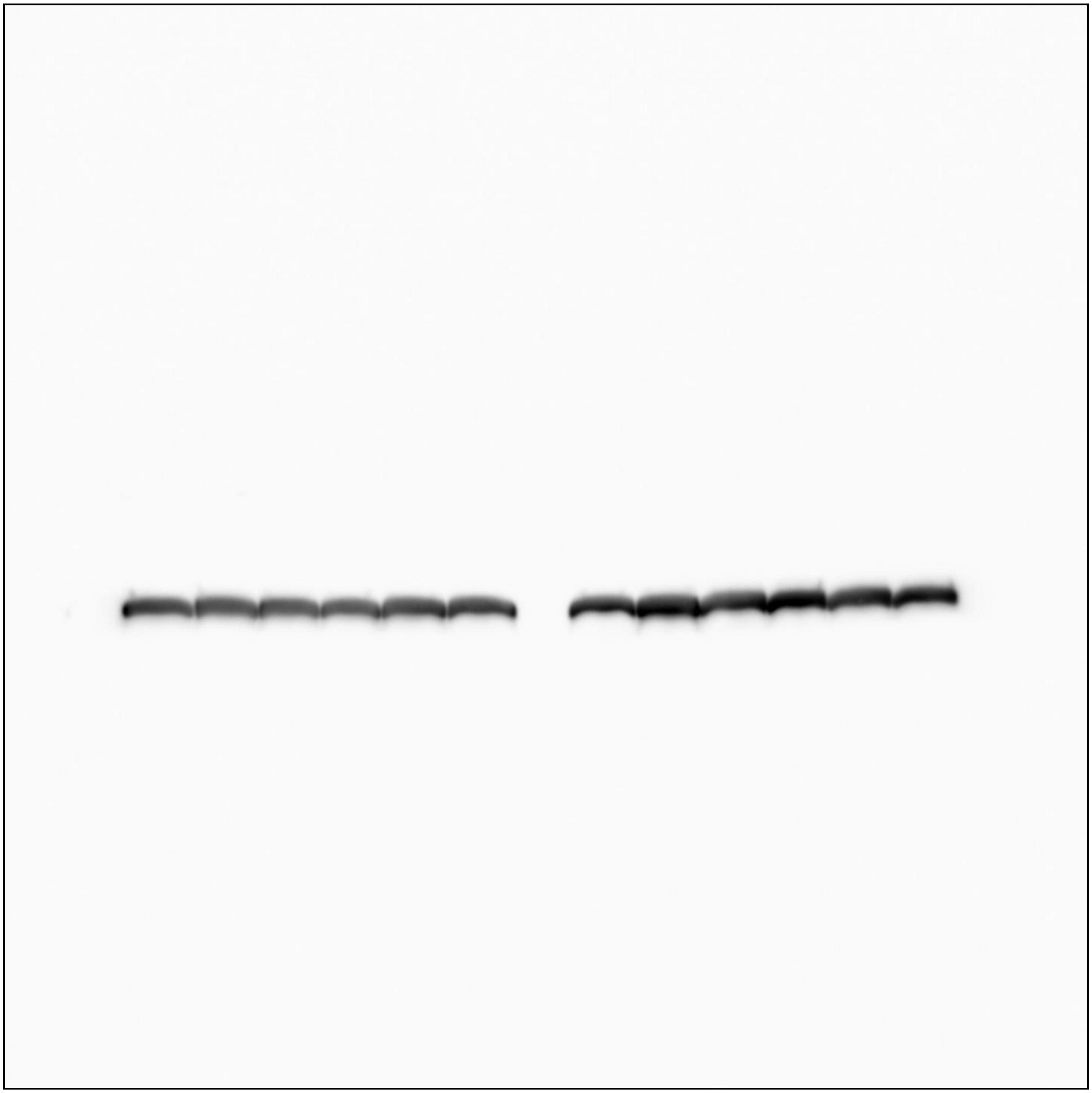

Sample 1 & 2: Plots of Tubulin

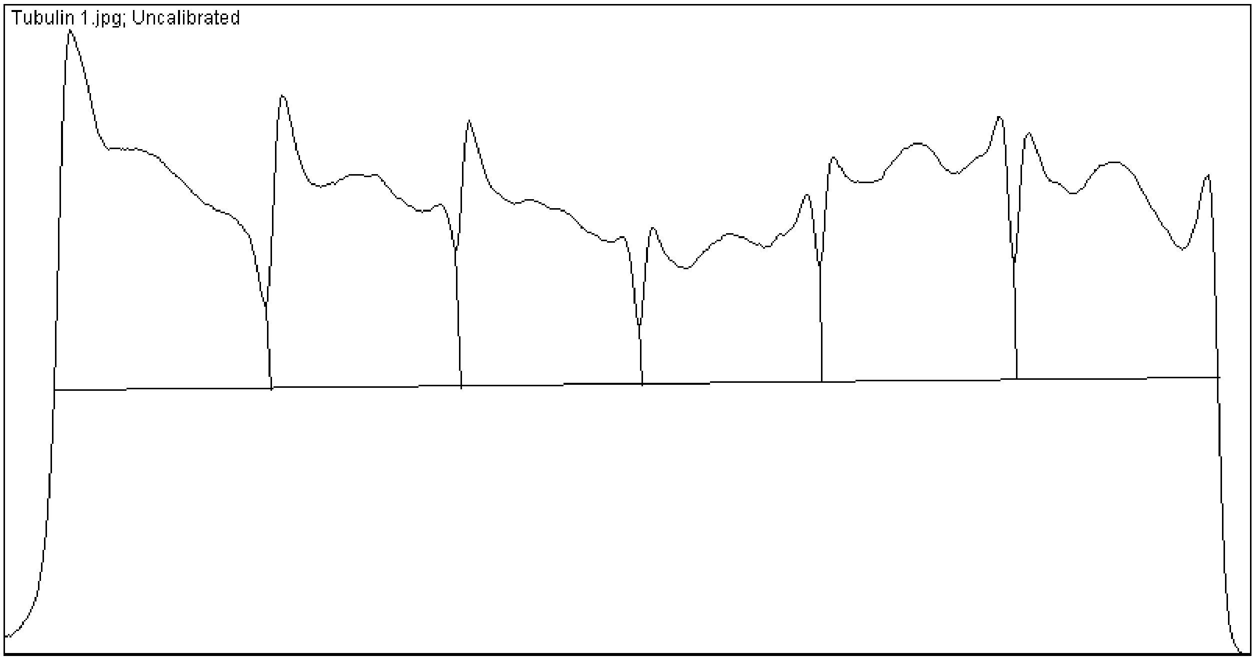

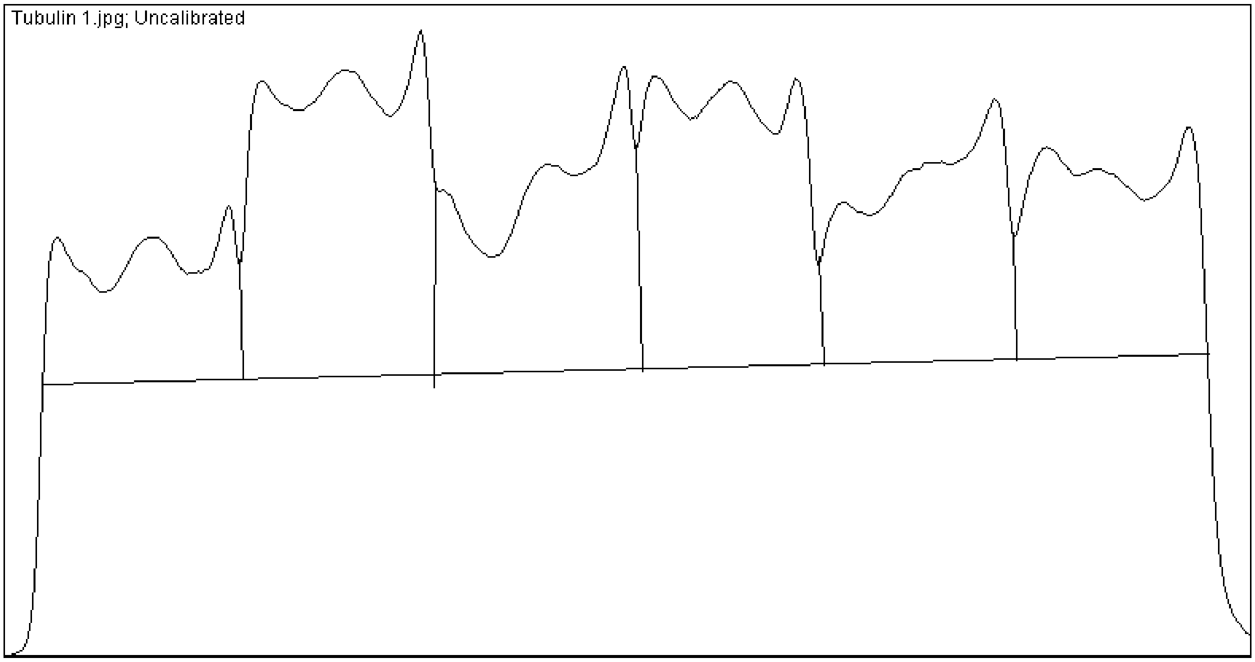

Sample 3 & 4: NLRP3 Blot

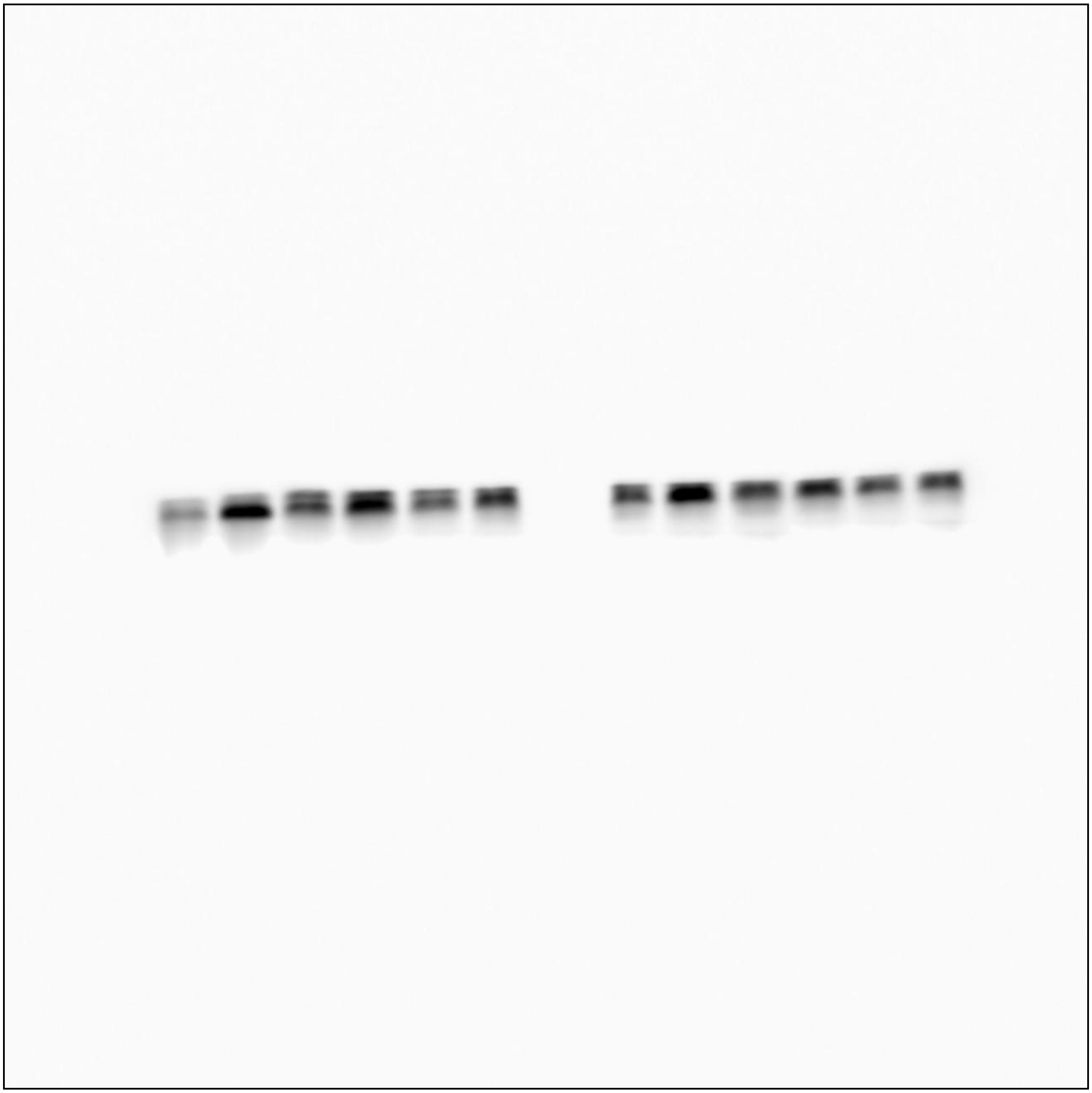

Sample 3 & 4: Plots of NLRP3

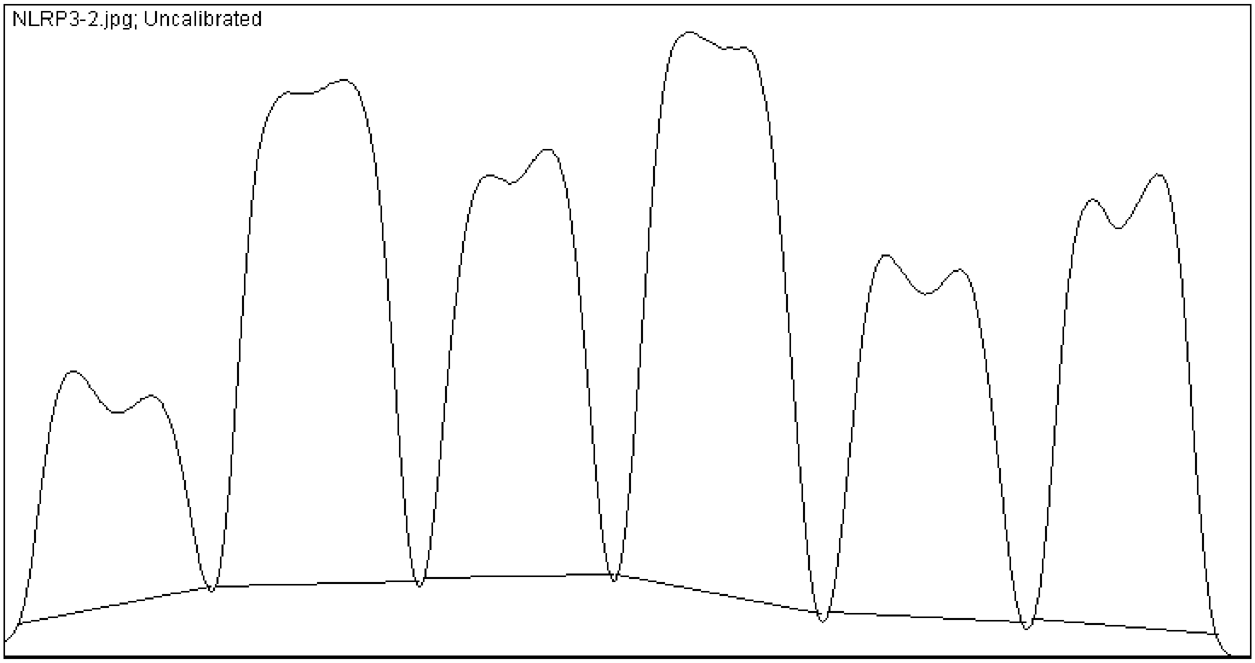

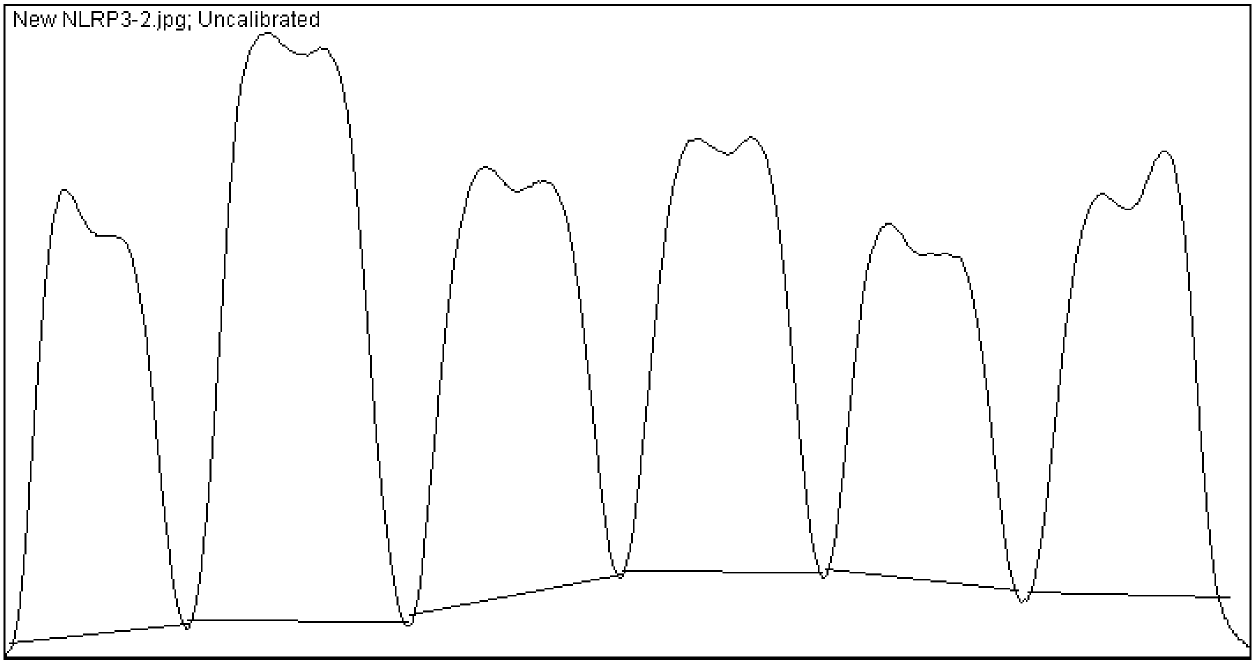

Sample 3 & 4: Tubulin Blot

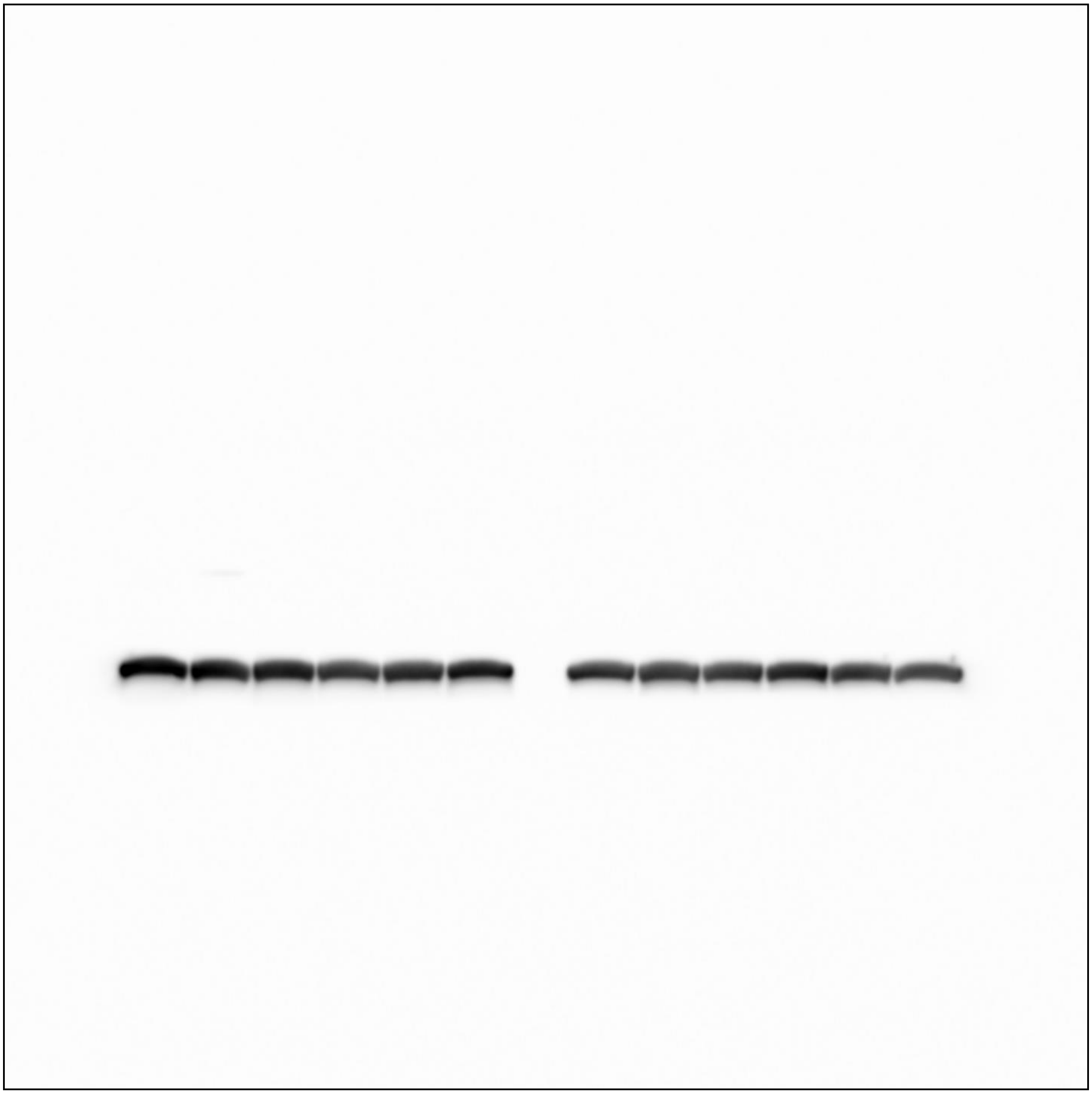

Sample 3 & 4: Plots of Tubulin

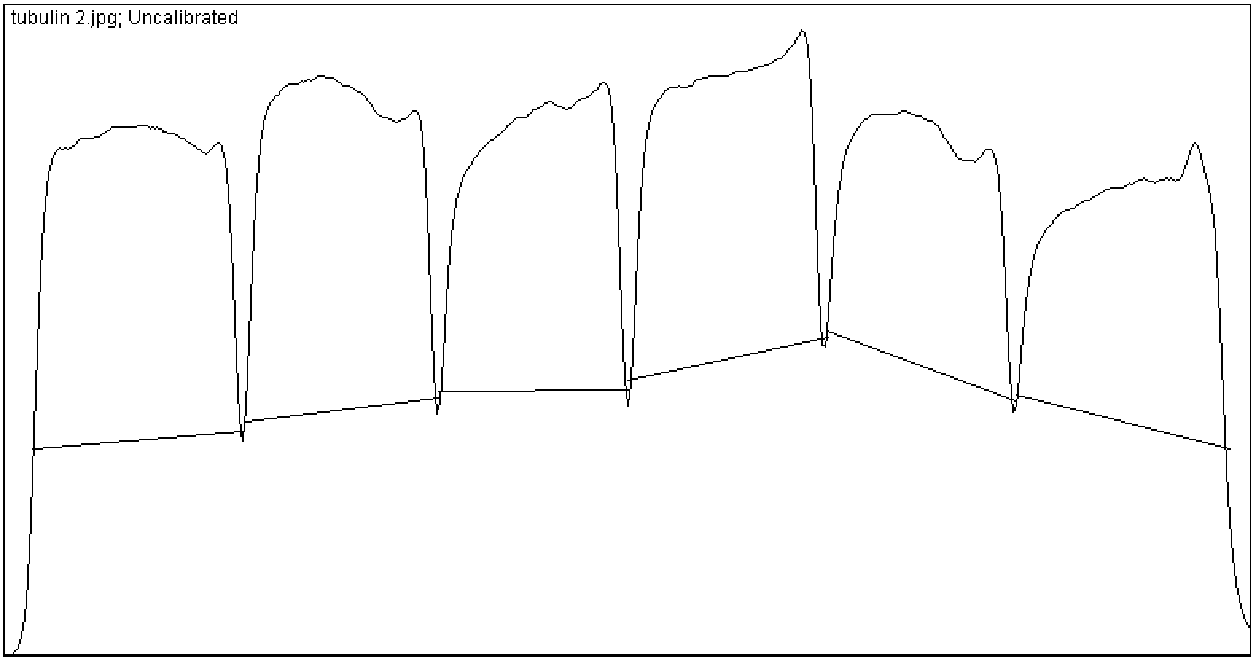

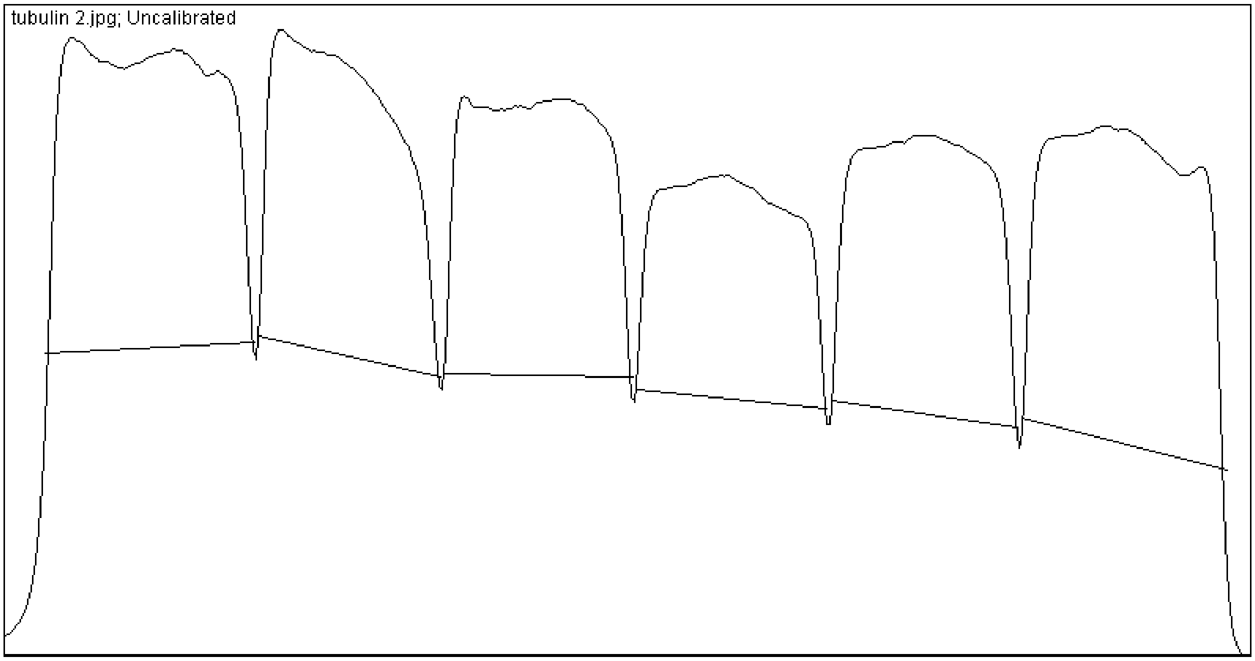

Sample 5: NLRP3 Blot

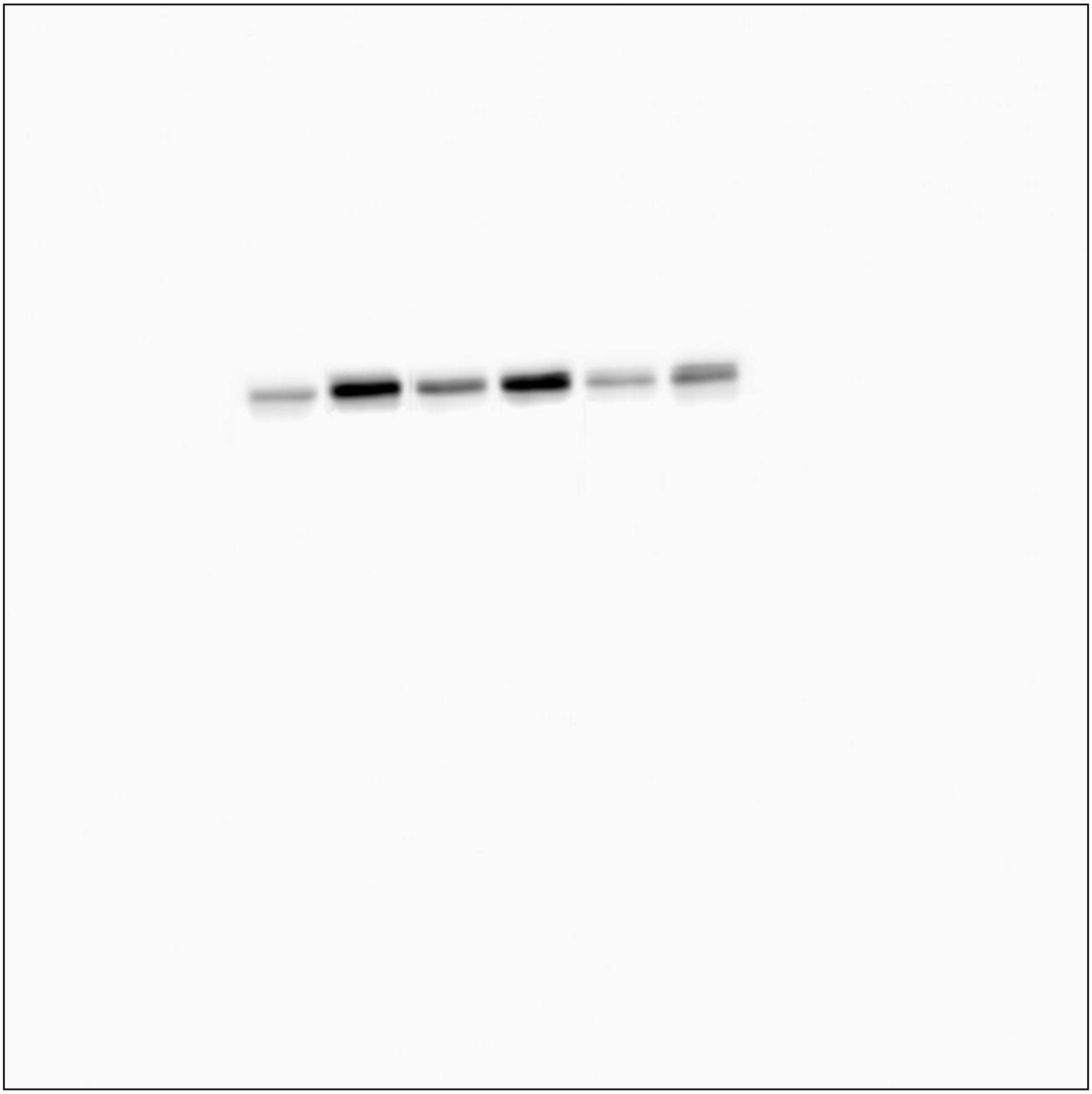

Sample 5: Plots of NLRP3

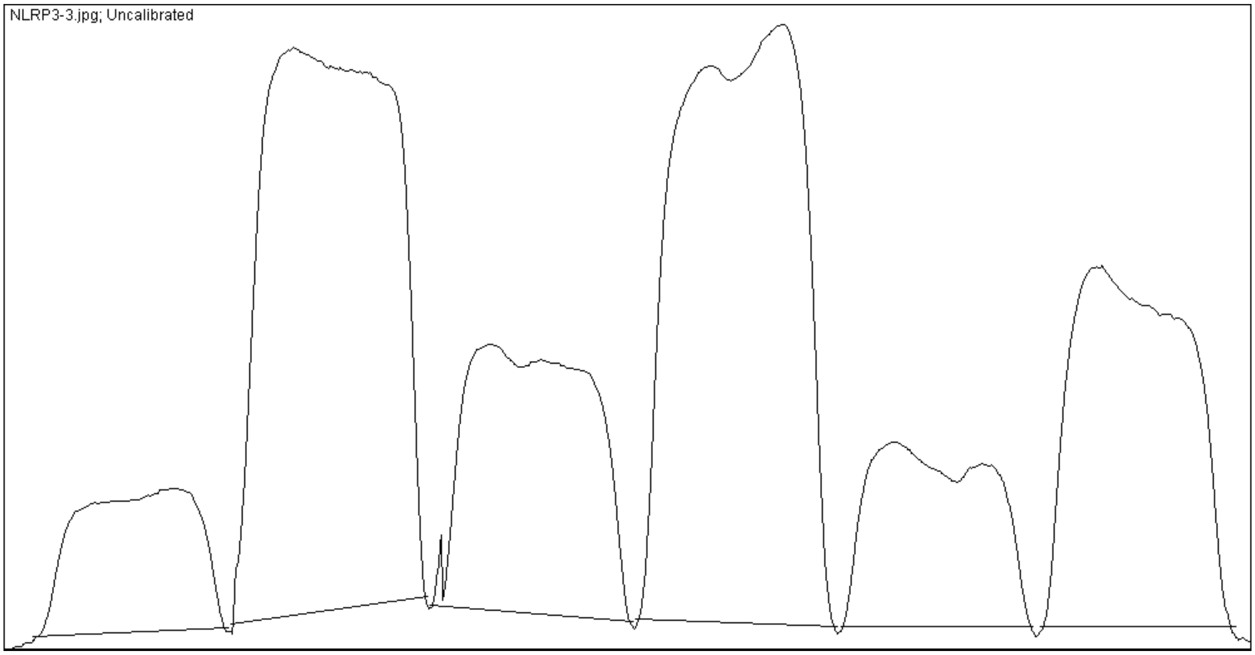

Sample 5: Tubulin Blot

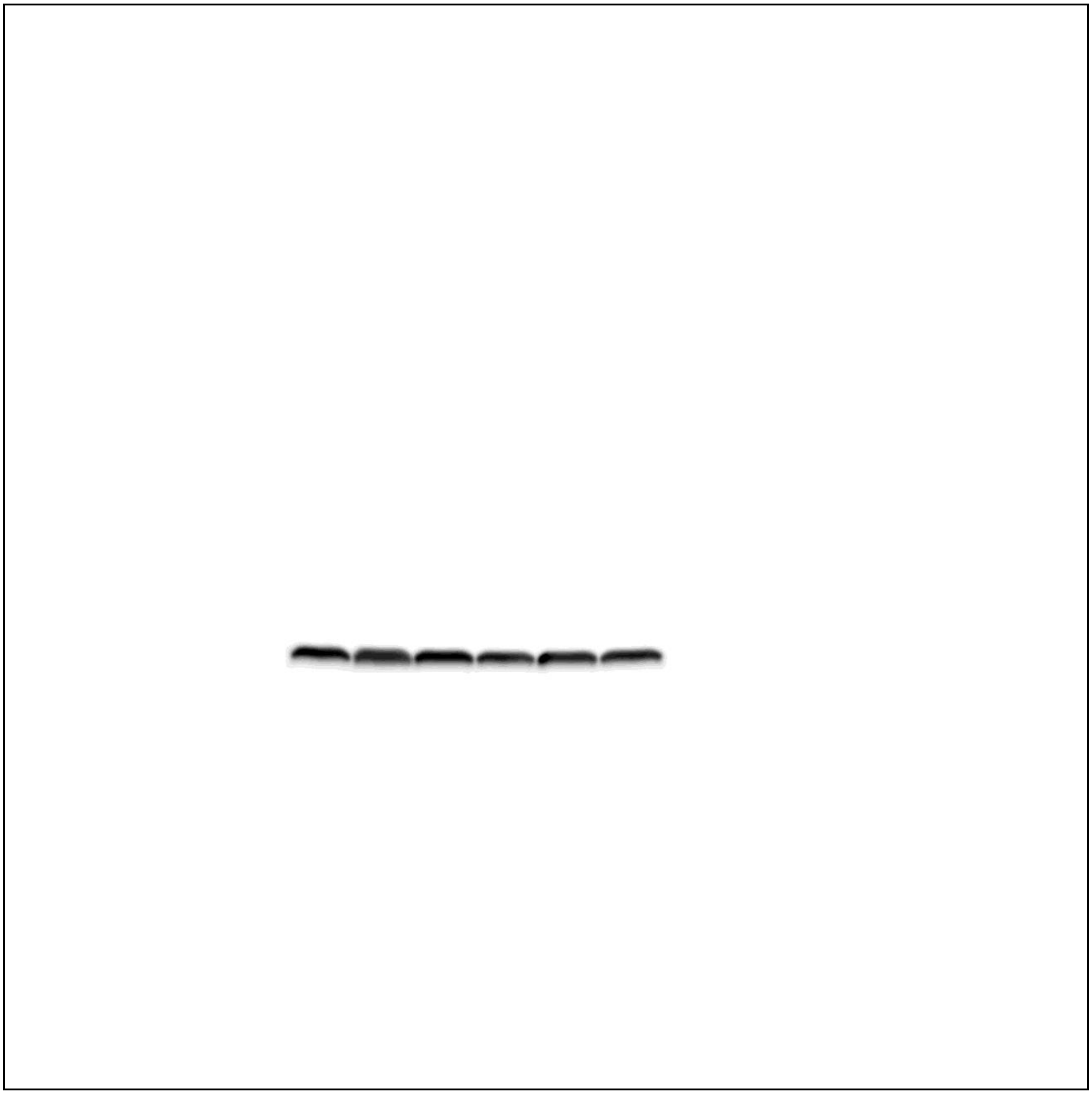

Sample 5: Plots of Tubulin

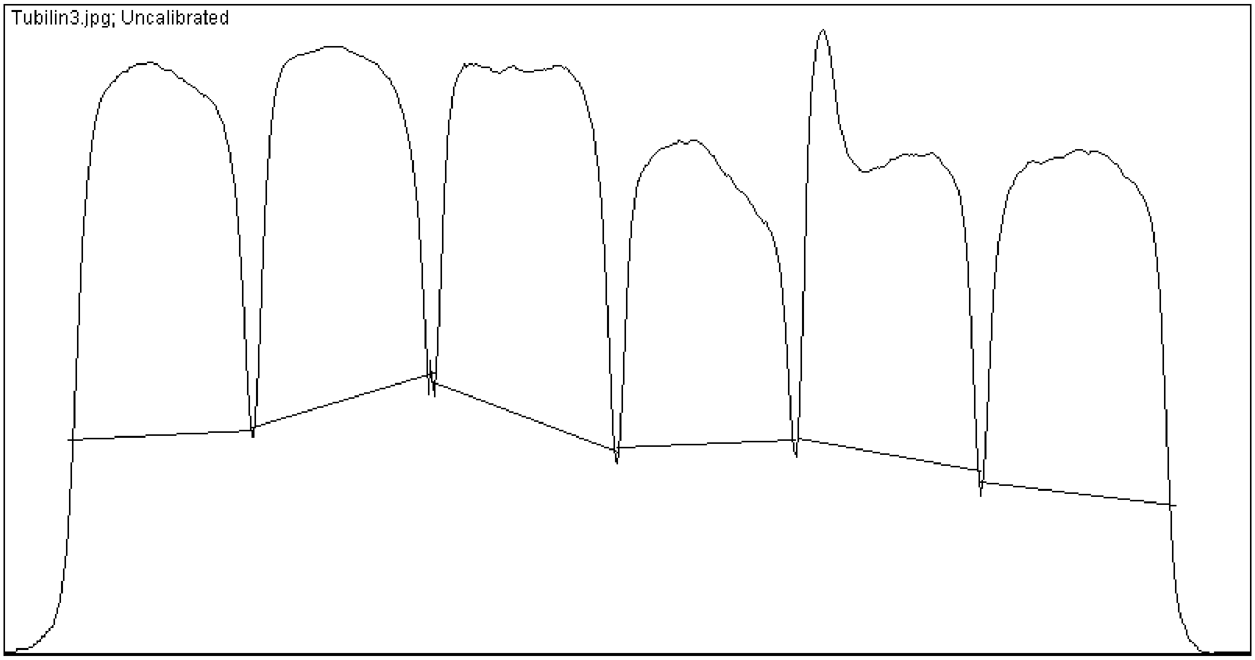

Sample 6: NLRP3 Blot

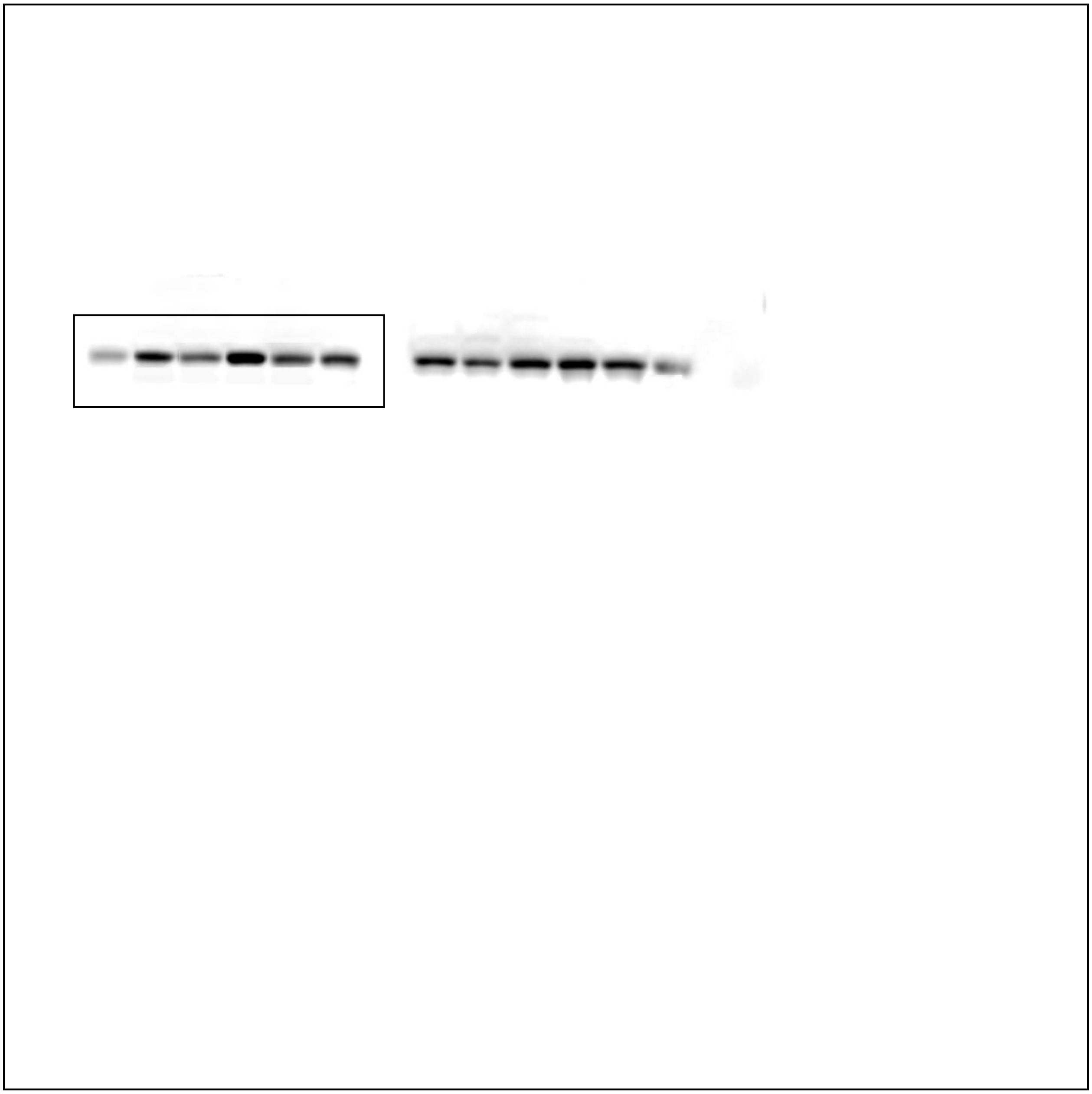

Note: The left side set of the blot (boxed) is considered

Sample 6: Plots of NLRP3

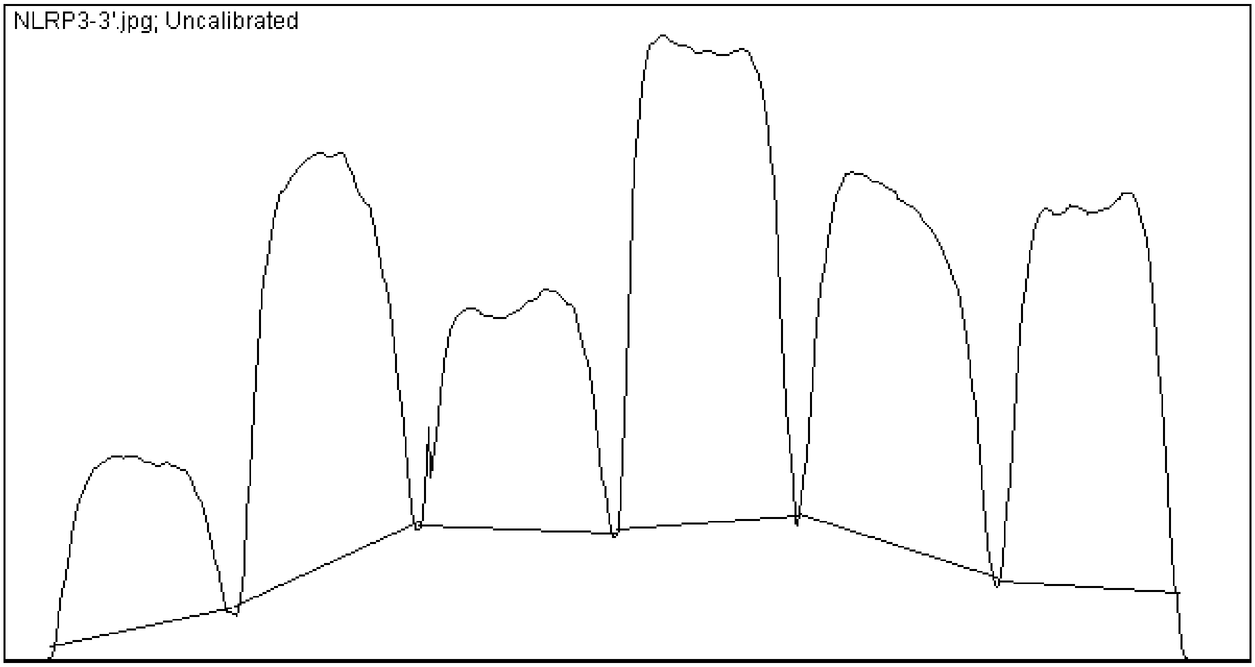

Sample 6: Tubulin Blot

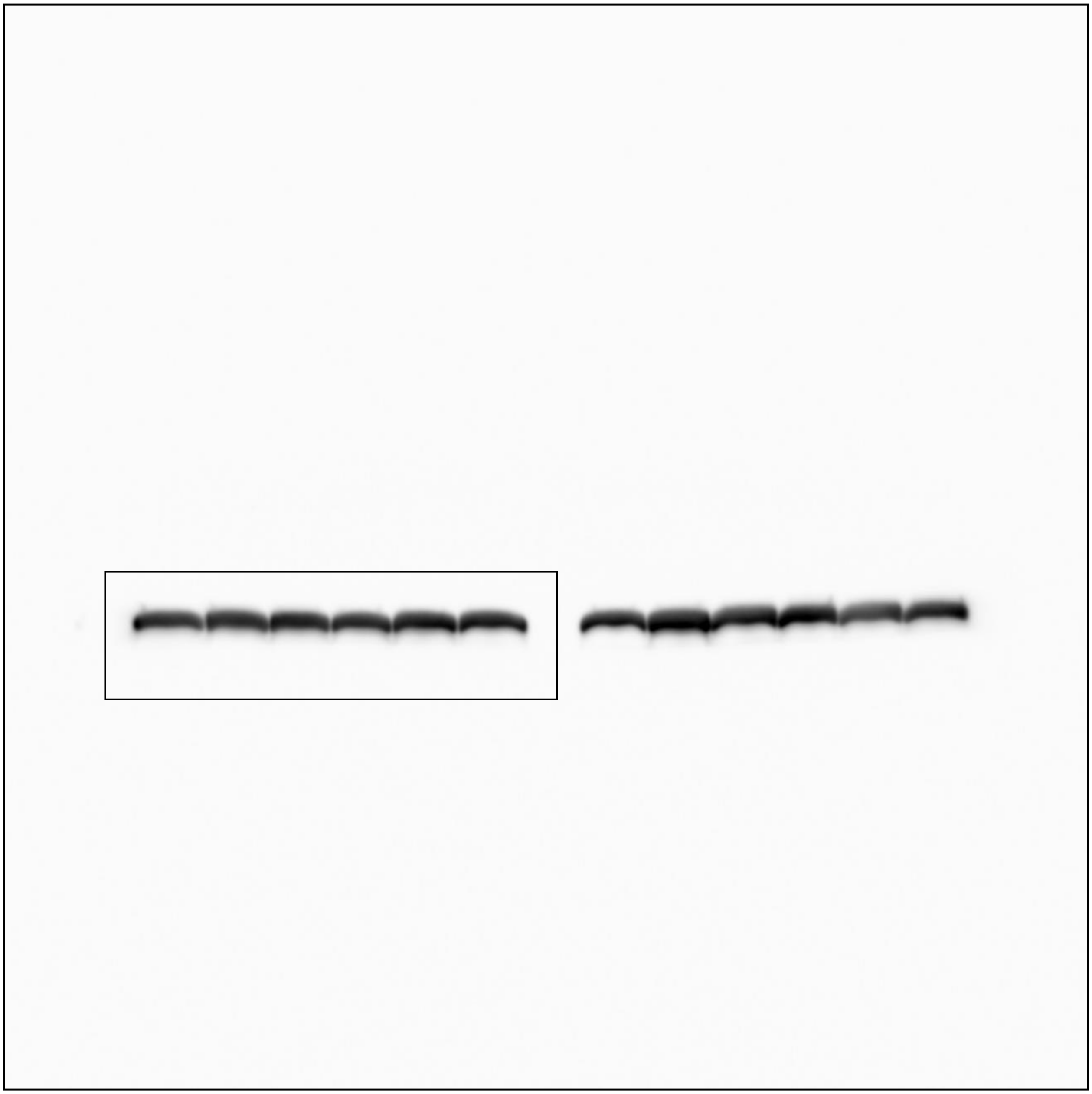

Note: The left side set of the blot (boxed) is considered

Sample 6: Plots of Tubulin

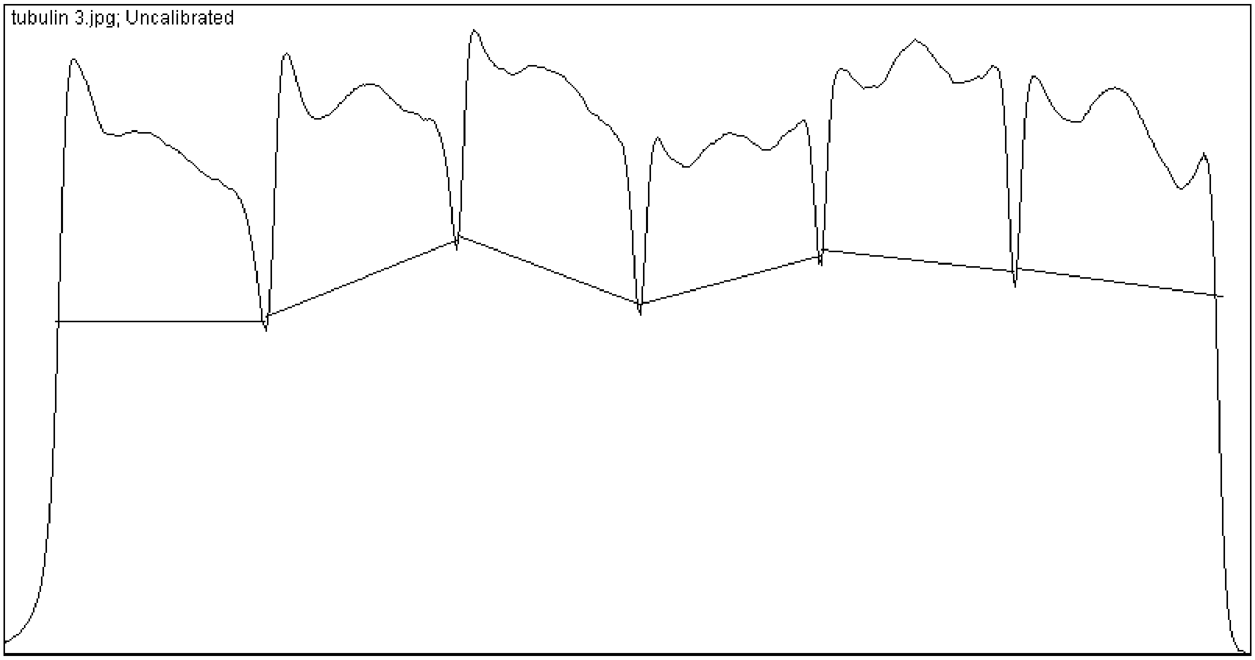

Supplement: S1 Data — (PDF) [file pone.0231543.s004.pdf]
